# Respiratory Syncytial Virus Vaccine Design Using Structure-Based Machine-Learning Models

**DOI:** 10.3390/v16060821

**Published:** 2024-05-22

**Authors:** Thomas C. McCarty, Iosif I. Vaisman

**Affiliations:** 1 RNA Viruses Section, Laboratory of Infectious Diseases, National Institute of Allergy and Infectious Diseases (NIAID), National Institutes of Health (NIH), Bethesda, MD 20892, USA; mccartyt@niaid.nih.gov; 2 School of Systems Biology, George Mason University, Manassas, VA 20110, USA

**Keywords:** negative-strand RNA virus, respiratory syncytial virus, interferon antagonist, computational analysis of viral protein structure, mutational analysis of interferon antagonist, machine learning, computational mutagenesis, live-attenuated virus vaccine design, viral protein structure modification, nonstructural protein 1, NS1

## Abstract

When designing live-attenuated respiratory syncytial virus (RSV) vaccine candidates, attenuating mutations can be developed through biologic selection or reverse-genetic manipulation and may include point mutations, codon and gene deletions, and genome rearrangements. Attenuation typically involves the reduction in virus replication, due to direct effects on viral structural and replicative machinery or viral factors that antagonize host defense or cause disease. However, attenuation must balance reduced replication and immunogenic antigen expression. In the present study, we explored a new approach in order to discover attenuating mutations. Specifically, we used protein structure modeling and computational methods to identify amino acid substitutions in the RSV nonstructural protein 1 (NS1) predicted to cause various levels of structural perturbation. Twelve different mutations predicted to alter the NS1 protein structure were introduced into infectious virus and analyzed in cell culture for effects on viral mRNA and protein expression, interferon and cytokine expression, and caspase activation. We found the use of structure-based machine learning to predict amino acid substitutions that reduce the thermodynamic stability of NS1 resulted in various levels of loss of NS1 function, exemplified by effects including reduced multi-cycle viral replication in cells competent for type I interferon, reduced expression of viral mRNAs and proteins, and increased interferon and apoptosis responses.

## 1. Introduction

Vaccines play a major role in controlling viral disease. A number of the most successful viral vaccines have been live-attenuated vaccines. The development of live-attenuated viral vaccines can be a painstaking trial-and-error process of reiterative testing to obtain derivatives with suitable levels of attenuation and efficacy. Attenuation typically involves mutations that reduce virus replication. This might involve direct effects on the activity or stability of viral components, such as ones involved in the viral replicative cycle, or indirect effects such as reducing the ability of the virus to counter the host immune response or cause disease. Complicating factors include the necessity that the reduction in viral replication associated with attenuation must not compromise the ability to grow the virus sufficiently well in vitro for manufacture, nor the ability to replicate sufficiently and retain satisfactory immunogenicity in the immunized host.

Computational biology has influenced numerous fields of biomedical research [1,2,3,4]. In particular, the field of protein folding/protein modeling has advanced dramatically due to synergistic improvements in both computational hardware and algorithms [5,6,7]. Machine learning can substantially assist reiterative processes such as vaccine design [8], providing flexibility in attenuation levels and, therefore, in the development of live-attenuated virus vaccines.

RSV is a nonsegmented negative-strand RNA virus of the *Orthopneumovirus* genus of the *Pneumoviridae* family in the *Mononegavirales* order. RSV is the most important viral agent of severe pediatric respiratory tract disease worldwide [9,10,11]. Recent estimates have shown that, in 2015, there were about 33 million cases of RSV-associated respiratory tract infections worldwide, with about 3 million hospitalizations, and 60,000 in-hospital deaths in children younger than 5 years [11]. High RSV-related morbidity and mortality presents a substantial burden on health-care services, both in industrialized and developing countries. Despite active ongoing research, RSV lacks a licensed live-attenuated vaccine suitable for use during routine newborn immunizations. The RSV genome consists of a single-stranded negative-sense RNA 15.2 kb in length that has 10 genes in the order 3′-NS1-NS2-N-P-M-SH-G-F-M2-L-5′, flanked by short 3′-terminal leader and 5′-terminal trailer regions [9]. The genes begin and end with short, conserved gene-start and gene-end transcription signals, respectively, and are transcribed as individual mRNAs by sequential transcription [12,13].

The RSV NS1 protein is unique to the *Orthopneumovirus* genus [14] and lacks known related counterparts in other human viruses. As the most promoter-proximal gene in the RSV genome, NS1 encodes the most abundantly expressed mRNA and protein during an RSV infection. RSV encodes a second NS protein called NS2: NS1 and NS2 interfere with a number of host cell responses and appear to do so acting both individually and as a metastable complex called the NS degradasome [15]. In particular, the NS proteins target multiple signaling cascades related to the induction and signaling of type I and III interferons (IFNs) [16,17,18,19,20,21,22] that are key for cells to establish an antiviral state. For example, the NS1 protein has been shown to associate with mitochondrial antiviral-signaling (MAVS) protein in A549 cells, preventing the interaction between retinoic acid-inducible gene I (RIG-I) and MAVS [23,24,25,26,27], an essential early step in the induction of an antiviral response. Additionally, possibly through a RIG-I—MAVS disruption, NS1 has been shown to prevent activation (translocation) of the interferon regulatory factor 3 (IRF3) and nuclear factor kappa-light-chain-enhancer of activated B cells (NFκB) transcription factors and IRF3- or NFκB-dependent expression of genes involved in antiviral activities including inflammation and apoptosis [22,28,29]. At the level of IFN-induced signaling, NS1 appears to disrupt signaling by the IFN-activated Janus kinases (JAK)–signal transducer and activator of transcription proteins (STAT) receptor complex. NS1 was shown to reduce signal transducer and activator of transcription 2 (STAT2) levels [30,31,32] as well as prevent its nuclear translocation [33]. With regard to replicating virus, RSV bearing the deletion of the NS1 gene retains the ability to replicate in vitro and in vivo [34], although at reduced levels in cells competent for type I IFN responses. NS1-deletion viruses are presently being evaluated in a clinical trial as candidate live vaccines (ClinicalTrials.gov Identifier: NCT03596801; RSV 6120/∆NS1 and RSV 6120/F1/G2/∆NS1, accessed on 19 May 2024) [35]. However, the complete deletion of NS1 was highly attenuating in animal models, and it would be desirable to have NS1 mutations that confer less attenuation.

The multiple roles of the NS1 protein in antagonizing host defenses and promoting viral growth provide opportunities to quantify the effects of mutations, such as by measuring viral replication, viral gene expression, host defense gene expression, and apoptosis. The use of computational methods to identify amino substitutions that may affect NS1 protein structure and function to varying extents provides a new method towards obtaining a spectrum of attenuating mutations that may be useful for optimizing live-attenuated vaccine candidates. In the present study, we explored the novel approach of using structure-based computation methods to guide the generation of potential attenuating mutations. We applied machine learning to identify amino acid substitutions throughout the 3D protein structure of NS1 predicted to affect thermodynamic stability of the NS1 protein. These mutations provided a range of effects on NS1 protein function and represent new attenuating mutations.

## 2. Materials and Methods

### 2.1. In Silico Mutation Analysis by Machine Learning

AUTO-MUTE 2.0 (http://binf.gmu.edu/automute, accessed on 19 May 2024) [36,37] is a computational mutagenesis tool utilizing a combination of computational geometry and machine learning. Identifying the six closest amino acid residue positions (<12 angstroms away) surrounding a residue targeted for mutation, the software predicts effects of specific amino acid change(s) on the overall function and thermodynamic properties of a protein. An input feature vector including four-body, knowledge-based, statistical contact potentials regarding the original residue, the mutated residue, and the six closest neighbors [38] was used to calculate related residue replacement feature vectors. AUTO-MUTE 2.0 predictive models trained using large sets of sequence-dissimilar protein structures with diverse mutations and experimentally studied functional effects were used to classify these residue replacement feature vectors into predictive groups.

Among the different free energy change calculations and machine-learning classifiers available, we used stability_changes_dTm.pl because of its simple thermal stability prediction labels (Increased or Decreased) and overall superiority described in a previously published classifier comparison [37,39,40] to obtain a general categorization of all possible mutations. Additionally, we used stability_changes_ddG.pl to generate differential energy (ddG) prediction values for each possible substitution. Averaged ddG values among substitutions that belong to specific amino acid groups (increased interior or protein–protein interface frequency) [41] were used to highlight/identify specific substitution locations in the protein. The 3D structure of human RSV NS1 (PDB 5VJ2; https://www.rcsb.org/structure/5vj2, accessed on 19 May 2024) [17]) was used as input to AUTO-MUTE 2.0 and a single output file containing either the amino acid stability change effect prediction or ddG value prediction was returned as output (Files S1, S2 and Table S1). Elapsed real time for AUTO-MUTE 2.0 to make each type of prediction for all amino acid substitutions in 5VJ2 was about 72 h.

### 2.2. Recombinant RSV Viruses

All RSV viruses in this study were based on recombinant RSV strain A2 (GenBank: KT992094.1) [42] that had been further modified as previously described by deletion and modification of the downstream untranslated region of the SH gene [43] and by the addition of a gene encoding the enhanced green fluorescent protein (eGFP, Clontech, Mountain View, CA, USA) between the RSV P and M genes [20]. The mutations in the SH gene were shown previously to have little or no effect on the replication and gene expression of RSV wt and RSV-GFP viruses [20,43]; the latter virus was used in this study and was considered to have a wild-type (wt) phenotype. A derivative of the wt RSV-GFP virus from which the NS1 gene was deleted, RSV-GFP/∆NS1, was made in previous work [20]: apart from the deletion of the NS1 gene, these viruses are identical in terms of nucleotide sequence.

**Figure 1:**
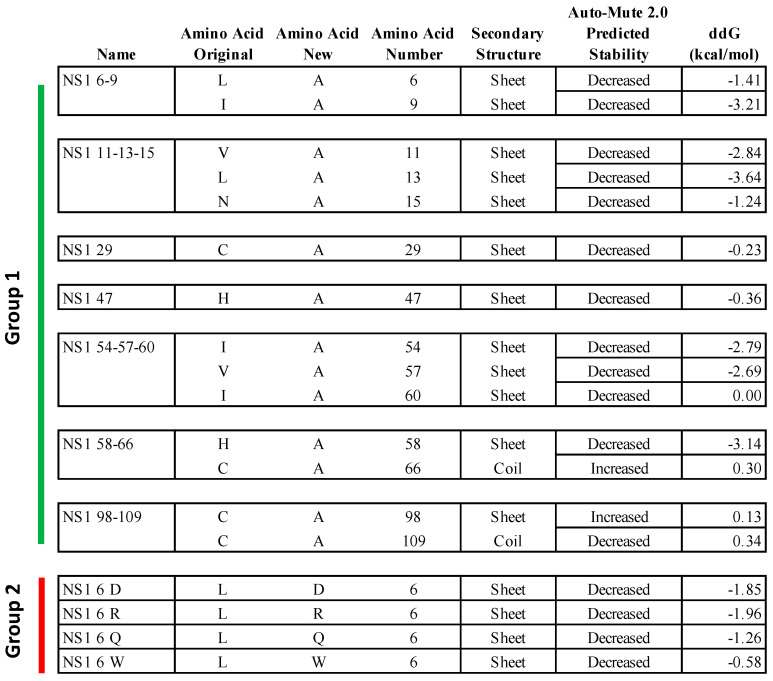
Predicted properties of amino acid substitutions in the 5VJ2 NS1 protein structure. Amino acid substitutions chosen based on the AUTO-MUTE 2.0 predicted effect in the NS1 protein structure and engineered into replicating virus. The figure includes the mutant virus names, original amino acid assignments, new mutant assignments, positions in the amino acid sequence, local secondary structure, predicted effect on stability, and predicted change in free energy (ddG) in the 5VJ2 structure. Groups 1 and 2 are identified by vertical bars on the left.

In the present study, 12 derivatives of the wt RSV-GFP virus were made containing mutations in the NS1 protein (Figure 1). Mutations were introduced into the NS1 gene by site-directed mutagenesis (QuikChange Lightning Site-Directed Mutagenesis Kit, Agilent Technologies, Santa Clara, CA, USA) of an RSV antigenomic cDNA subclone containing the leader, NS1 and NS2 sequence flanked by Not I and Kpn I restriction endonuclease sites. Mutated Not I—Kpn I fragments were used to replace the wild-type sequence in a similarly restriction-endonuclease-digested plasmid containing the full length antigenomic sequence of RSV-GFP. Complete-genome automated Sanger sequencing of all 12 mutant antigenomic cDNAs confirmed that the sequences were correct and free of adventitious mutations.

### 2.3. Cell Culture

BSR T7/5 cells, a baby hamster kidney 21 (BHK21) cell line that constitutively expresses T7 RNA polymerase, were maintained as previously described [44]. Vero cells (African green monkey kidney cell) (ATCC CCL-81, Manassas, VA, USA) and A549 cells (adenocarcinoma human alveolar basal epithelial cell) (ATCC CCL-185) were maintained as previously described [45,46].

### 2.4. Virus Recovery by Reverse Genetics

Mutant viruses were recovered by reverse genetic methods as previously described [42,47]. Briefly, each antigenomic cDNA was transfected with helper plasmids expressing the RSV N, P, M2-1, and L proteins into BSR T7/5 cells. Twenty-four hours post transfection, the cells were scraped into the medium, transferred to Vero cells, supplemented with additional medium, incubated for 3–5 days until cytopathic effect was extensive, and harvested to yield passage 1 (P1) virus. Viruses were passaged twice in Vero cells using multiplicity of infection (MOI) of 0.1 plaque-forming units per cell (pfu/cell) to obtain P3 stocks that were used in all experiments; complete-genome sequences were confirmed by automated Sanger sequencing of uncloned RT-PCR produces as previously described [48].

### 2.5. Virus Titration and Multicycle Replication Assay

**Figure 2:**
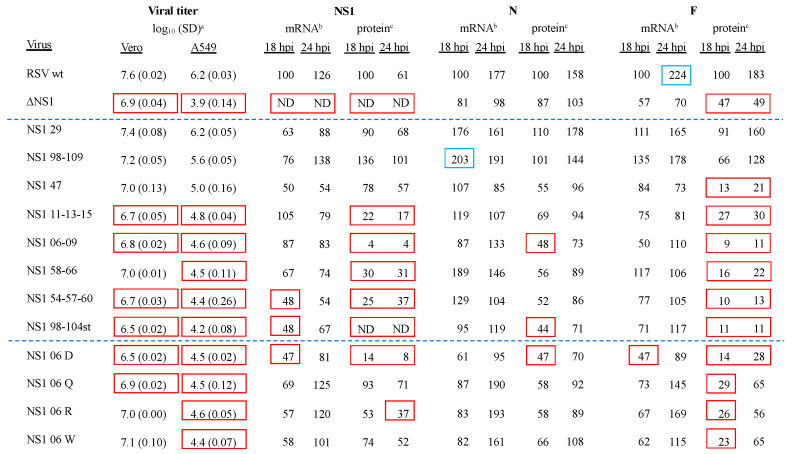
NS1 substitution virus growth in Vero and A549 cells; mRNA and protein expression in A549 cells. ^a^ Log_10_ peak virus growth, Vero on Day 4, and A549 on Day 3 (standard deviation SD, in parenthesis). Red boxes indicate <7.0 log_10_ in Vero cells or <5.0 log_10_ in A549 cells. ^b^ RSV NS1, N, and F mRNA expression. Red and blue boxes indicate <50% and >200%, respectively, of RSV wt expression at 18 hpi. ^c^ RSV NS1, N, and F protein expression normalized to endogenous GAPDH. Red boxes indicate <50% of RSV wt expression at 18 hpi. ND—Not detected. Dashed blue lines separate the control virus results (top) from Group 1 viruses (middle) and Group 2 viruses (bottom).

Virus titrations were performed by plaque assay in 24-well plates of Vero cells infected with ten-fold serial dilutions of virus and incubated under an 0.8% methyl cellulose overlay at 37 °C [49]. Plaques were visualized and counted based on GFP expression using an Amersham Typhoon Imaging System (GE Healthcare Life Sciences, Chicago, IL, USA) and ImageJ (Version 1.52d NIH/Wayne Rasband; https://imagej.nih.gov/ij, accessed on 19 May 2024) and expressed as plaque-forming units per ml (pfu/mL). To measure multicycle replication, replicate monolayer cultures of Vero and A549 cells in 12-well plates were infected with the NS1 mutant viruses, wt RSV-GFP, and RSV-GFP/∆NS1 at an MOI of 0.1 pfu/cell, and incubated at 37 °C for 7 days. At 24 h intervals, one well per virus and time point was scraped into the medium and briefly vortexed, and clarified medium supernatants were analyzed by virus titration as described above. The multicycle replication experiment was performed two or three times for the various mutants (Figure 2; Tables S2–S4).

### 2.6. Quantitative RT-PCR (qRT-PCR)

**Figure 3:**
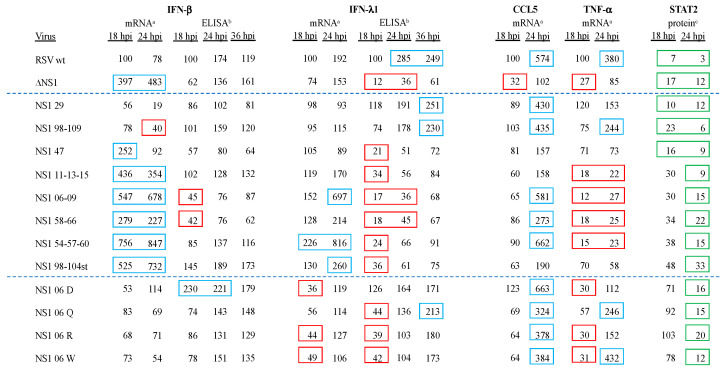
Host mRNA and protein expression during NS1 substitution virus infection in A549 cells. ^a^ A549 host cell mRNA expression. Red box indicates <50%; blue box indicates >200%; and green box indicates <25%, respectively, of RSV wt expression at 18 hpi. ^b^ Growth media concentrations of IFN-β and IFN-λ1 calculated from standard curves. Red and blue boxes indicate <50% and >200%, respectively, of the RSV wt expression at 18 hpi. ^c^ A549 cell STAT2 protein expression normalized to endogenous GAPDH. Green box indicates >25% of STAT2 protein in uninfected A549 cells at 18 hpi. Dashed blue lines separate the control virus results (top) from Group 1 viruses (middle) and Group 2 viruses (bottom).

Replicate monolayer cultures of A549 cells in 12-well plates were infected with the indicated RSV at an MOI of 3.0 pfu/cell. One well per virus and time point was harvested at 18 and 24 h post infection (hpi), and total cell-associated RNA was isolated with RNeasy Mini Kit (Qiagen, Gaithersburg, MD, USA) as recommended by the manufacturer. A fixed volume of total RNA was combined with a specific TaqMan Gene Expression Assay using the TaqMan RNA-to-Ct 1-Step Kit (Life Technologies, Frederick, MD, USA) as recommended by the manufacturer. Four replicate qRT-PCR reactions were performed for each sample. The TaqMan gene expression assay 18S rRNA (Hs99999901_s1) was used as a normalization control in conjunction with the following TaqMan assays IFNB1 (Hs01077958_s1), IFNL1 (Hs00601677_g1), CCL5 (Hs00174575_m1), and TNF-α (Hs00174128_m1), or custom-designed TaqMan assays RSV NS1, RSV N, and RSV F (Table S5). TaqMan Gene Expression assay reactions were analyzed on the 7900HT Fast Real-Time PCR system (Applied Biosystems, Foster City, CA, USA) as previously described [46]. Each RNA was analyzed in three experiments (Figure 2 and Figure 3; Tables S6 and S7).

### 2.7. Western Blot

Replicate monolayer cultures of A549 cells in 12-well plates were infected with the indicated RSV at an MOI of 3.0 pfu/cell. One well per virus and time point was harvested at 18 and 24 hpi and used to prepare Western blots as previously described [46]. RSV NS1, N, and F proteins were detected with mouse monoclonal antibody (mAb) 1E-5-1 (HHS Reference E-167-2018-0, Kerafast, Shirley, MA, USA) mouse mAb ab94806 (Abcam, Cambridge, UK), and mouse mAb ab43812 (Abcam), respectively. STAT2 and glyceraldehyde-3-phosphate dehydrogenase (GAPDH) proteins were detected with mouse mAb sc-1668 (Santa Cruz Biotechnology, Dallas, TX, USA) or an anti-rabbit polyclonal antibody 10494-1-AP (Proteintech, Rosemont, IL, USA), respectively. Secondary antibodies were infrared dye-conjugated goat anti-mouse immunoglobulin 800CW and goat anti-rabbit immunoglobulin 680RD (Li-Cor, Lincoln, NE, USA). Western blot images were acquired on the Odyssey infrared scanner (Li-Cor) and analyzed with Image Studio Software (Version 5.2.5, Li-Cor). Each protein was analyzed in three experiments (Figure 2 and Figure 3; Figures S1–S4; Tables S8–S11).

### 2.8. ELISA

Replicate monolayer cultures of A549 cells in 12-well plates were infected with the indicated RSV at an MOI of 3.0 pfu/cell. Culture media from one well per virus and time point was harvested at 18, 24, and 36 hpi and clarified by low-speed centrifugation. The concentration of IFN-β was measured using the VeriKine Human IFN Beta ELISA Kit (PBL) and IFN-λ1 was measured using the Human IL-29 ELISA Kit (Invitrogen, Waltham, MA, USA) as recommended by the manufacturer and previously described [20]. ELISA plate measurements were recorded using a Synergy 2 Multi Mode Microplate Reader (BioTek, Winooski, VT, USA) as recommended by the manufacturer. Standard curves were generated using a 4-parameter fit in the Gen5 software (Version 3.04, BioTek) as recommended by the manufacturer. Each protein was analyzed in three experiments (see Figure 3; Table S12).

### 2.9. Caspase 3/7 Assay

**Figure 4:**
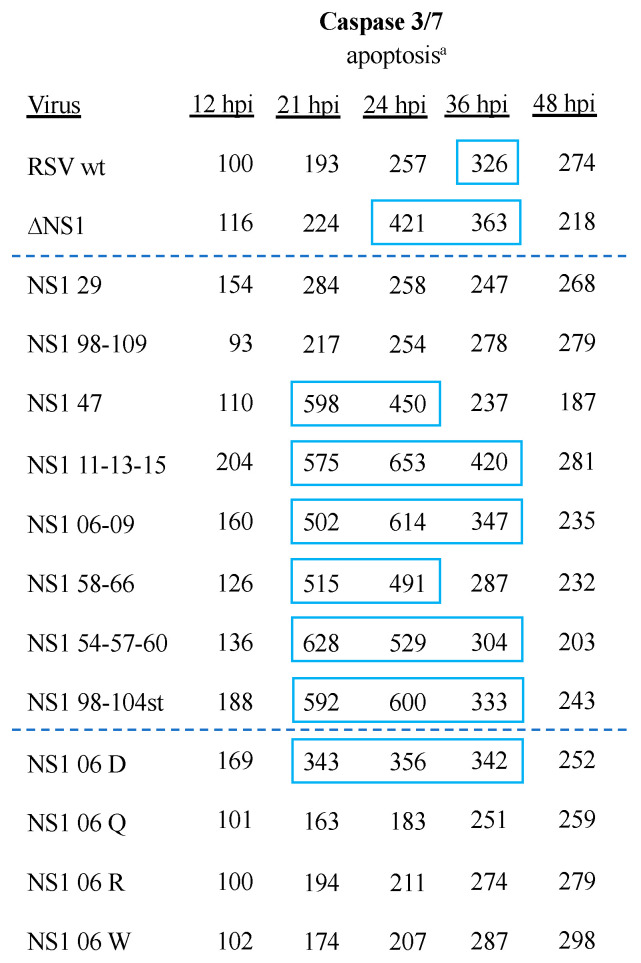
Caspase 3/7 activity in A549 cells infected with NS1 substitution viruses. ^a^ A549 caspase 3/7 activity. Blue box indicates >300% of RSV wt activity at 12 hpi. Dashed blue lines separate the control virus results (top) from Group 1 viruses (middle) and Group 2 viruses (bottom).

Replicate monolayer cultures of A549 cells in 96-well plates (PS F-Bottom Cell Culture Microplate—655090; Greiner Bio-One, Kremsmunster, Austria) were infected with each indicated RSV at an MOI of 3.0 pfu/cell (96 well) and incubated at 37 °C. The cultures were assayed for caspase activity at 12, 21, 24, 36, and 48 hpi using the Caspase-Glo 3/7 Assay (Promega, Madison, WI, USA) as recommended by the manufacturer. Each virus/time point was measured with quadruplicate wells. Caspase activity measurements were recorded using a Synergy 2 Multi Mode Microplate Reader (BioTek) as recommended by the manufacturer. The experiment was performed twice (see Figure 4; Table S13).

## 3. Results

### 3.1. Predicted Effects of Amino Acid Substitutions in the NS1 Protein Using the AUTO-MUTE 2.0

The use of machine-learning models to predict potential promising mutations in the NS1 protein required a 3D protein structure. An experimentally determined structure of RSV NS1 protein was not available when this study was initiated; therefore, we used the protein structure prediction program I-TASSER to generate a 3D ab initio structure of the 139 amino acid NS1 protein, resulting in a protein structure model called Model 1 as an initial structure surrogate input for machine-learning models of mutant stability prediction. However, an X-ray crystal structure of NS1 (PDB 5VJ2) was reported [17] while this work was in progress. The experimental structure turned out to be significantly different from our model, but the stability predictions from the model and the experimental structure were correlated reasonably well. In this paper, we report the results obtained using the experimental structure only. The similarities and differences between predictions based on an imperfect model and the X-ray structure, which might be of interest for the cases when an experimental structure is not available, will be discussed elsewhere.

The stability_changes_dTm.pl program in AUTO-MUTE 2.0 was used to predict Increased or Decreased thermodynamic stability associated with individual amino acid changes [37]. All possible amino acid replacements for all 138 of 139 residues in Model 1 of the NS1 protein were analyzed, excluding residue 68, which did not satisfy the six “nearest neighbors” requirement. The same thermodynamic stability predictions for all 135 amino acid replacements in the 5VJ2 structure were similarly calculated. The majority of these changes were predicted as Decreased (Model 1: 2036/2621 Decreased, 78%; 585/2621 Increased, 22%; 5VJ2: 1979/2565 Decreased, 77%; 586/2565 Increased, 23% (Files S1 and S3; Table S1); which reflects a known computational bias found among protein stability prediction programs [50,51]. In addition, specific ddG values were predicted using the stability_changes_ddG.pl program in AUTO-MUTE 2.0 to generate predicted ddG values (File S2). These ddG values represent the predicted change in free energy of the structure after each amino acid substitution, where negative or positive values are associated with reduced or increased protein stability, respectively. A comparison of predicted ddG values between Model 1 and 5VJ2 among shared amino acid substitutions revealed a moderate level of similarity. Eighteen specific mutations at 15 different amino acid positions of the NS1 protein sequence were chosen for experimental evaluation based on the overall predicted sensitivity (Decreased), average ddG predicted values among residue groupings (frequency found within the protein structure interior or protein–protein interfaces), and secondary structure location. Twelve RSV mutants were constructed containing one or more substitutions in the NS1 protein sequence of RSV-GFP (Figure 1).

Eight of these mutants were collectively called Group 1 (Figure 1). In the first seven of the Group 1 mutants, 1 to 3 amino acid assignments per virus were changed to alanine (A)—specifically, residues 6 and 9 (NS1 06-09); residues 11, 13, and 15 (NS1 11-13-15); residue 29 (NS1 29); residue 47 (NS1 47); residues 54, 57, and 60 (NS1 54-57-60); residues 58 and 66 (NS1 58-66); and residues 98 and 109 (NS1 98-109). The eighth mutant in Group 1, called NS1 98-104st, was inadvertently created due to a PCR error: in this virus, amino acid 98 was correctly changed to A, but, in addition, amino acids 101, 102, 103, and 104 were also changed (P to L, N to M, G to V, and L to Y) followed by the introduction of a stop codon after amino acid 104. Note that this last mutation is not shown in Figure 1.

In the remaining four RSV mutants, collectively called Group 2 (Figure 1), the leucine (L) residue at amino acid position 6 (6L) was replaced separately with four amino acids predicted to decrease protein thermal stability and with substantially different biochemical properties as compared to Leucine: namely, 6L was replaced with aspartic acid—6D (NS1 06 D), glutamine—6Q (NS1 06 Q), arginine—6R (NS1 06 R), or tryptophan—6W (NS1 06 W), which changed the non-polar (L) to either polar-charged (D), neutral polar (Q), charged aliphatic (R), or non-polar aromatic (W) amino acids.

**Figure 5:**
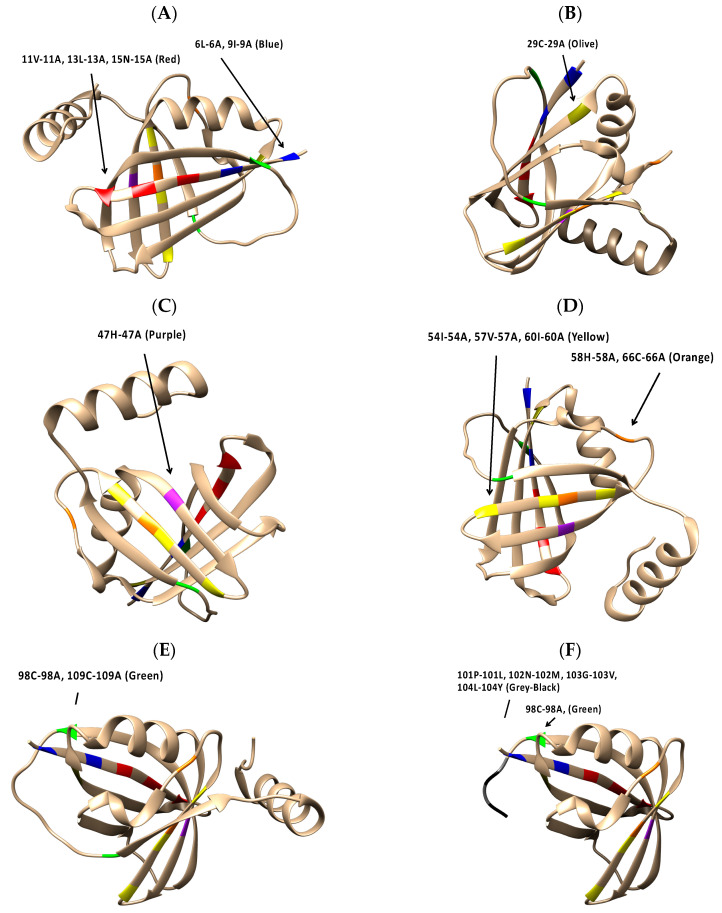
The locations of NS1 substitution mutations in the RSV NS1 (5VJ2) structure: (**A**) NS1 06-09 and NS1 11-13-15; (**B**) NS1 29; (**C**) NS1 47; (**D**) NS1 54-57-60 and NS1 58-66; (**E**) NS1 98-109; and (**F**) NS1 98-104st.

Figure 5A–F show the highlighted location of each amino acid substitution in the 5VJ2 protein structure. Among the Group 1 viruses, all substitutions are in sheet secondary structures. At approximately the midpoint in the amino acid sequence, NS1 58-66 (Figure 5D) straddles a sheet structure and an adjacent coil region in 5VJ2. Lastly, NS1 98-109 and NS1 98-104st (Figure 5E,F), situated in the last third of the NS1 protein, are located at opposite ends of a coil structure connecting two sheet strands in 5VJ2.

Figure 1 shows the AUTO-MUTE 2.0 thermodynamic stability and ddG value prediction for each amino acid substitution in 5VJ2 (not including mutant NS1 98-104st). Among the Group 1 viruses NS1 06-09 and NS1 11-13-15, the mutations at all five positions in 5VJ2 were predicted to result in decreased stability and negative ddG scores (Figure 1). In the remaining Group 1 viruses (NS1 29, NS1 47, NS1 54-57-60, NS1 58-66, and NS1 98-109), all contain a prediction of Decreased stability for 5VJ2 except for position 66 and 98. Within this same subset of substitutions, the association between the predictions of decreased or increased stability and negative or positive ddG scores seemed less robust in terms of the ddG magnitude and direction from 0. In the case of the Group 2 mutants, all the substitutions have decreased stability, and all were associated with negative ddG scores.

### 3.2. Multicycle Replications of RSV NS1 Substitution Viruses in Vero and A549 Cells

Multicycle virus growth was measured in Vero and A549 cells, which, respectively, do not and do produce type I IFNs in response to viral infection. Replicate monolayers of Vero and A549 cells were infected with the mutant viruses, in parallel with RSV-GFP and RSV-GFP/∆NS1 control viruses, at an MOI of 0.1 pfu/cell and incubated at 37 °C for 7 days. At 24 h intervals, cells were scraped into the medium and briefly vortexed, and the clarified medium supernatants were analyzed by plaque assay in Vero cells. Three (Group 1 viruses) or two (Group 2 viruses) separate experiments were performed, and the mean peak titer for each virus (which occurred on day 4 in Vero cells and day 3 in A549 cells) are shown for each cell line in Figure 2.

In Vero cells, the deletion of the NS1 gene resulted in a marginal (five-fold, 0.7 log_10_) decrease in replication (Figure 2), whereas, in A549 cells, the absence of the NS1 protein resulted in a much greater (200-fold, 2.3 log_10_) decrease in replication (Figure 2), as has been shown in numerous laboratories [16,34,52]. Previous studies showed that this reduction reflects restriction by host factors otherwise antagonized by NS1, such as the induction of IFN and apoptosis, and a comparison of mutant RSV virus replication in these two cell lines provides an assessment of NS1 function as a host response antagonist.

In Vero cells, Group 1 viruses NS1 29 and NS1 98-109 exhibited wt-like growth titers (7.4 and 7.2 log_10_, respectively). The remaining Group 1 members, as well as the Group 2 members, exhibited decreases of between 4- and 12-fold (0.6–1.1 log_10_) (Figure 2). For Group 1 in A549 cells, only NS1 29 exhibited viral growth similar to RSV wt (Figure 2). The remaining members of Group 1, as well as Group 2 members, all showed significant reductions in virus growth ranging from 25- to 100-fold lower (Figure 2). The Group 1 viruses are listed in Figure 2 and subsequent tables in the order of decreasing titer in A549 cells.

### 3.3. Viral mRNA and Protein Expression in A549 Cells Infected with RSV NS1 Substitution Viruses

We analyzed the expression of the viral NS1, N, and F mRNAs and proteins during infection of A549 cells with the mutant viruses. A549 cells were inoculated with the 12 mutant viruses in parallel with RSV-GFP and RSV-GFP/∆NS1 at an MOI of 3.0 pfu/cell, incubated at 37 °C, harvested at 18 and 24 hpi, and processed to prepare total cell-associated RNA or protein. The NS1, N, and F viral mRNAs were quantified by qRT-PCR, with each sample analyzed in quadruplicate. The mean of each quadruplicate was normalized relative to endogenous 18S rRNA and was expressed as a percentage of the normalized value of the same mRNA in RSV wt-infected cells at 18 hpi in the same experiment. Three separate experiments were performed, and the mean values are shown in Figure 2. The NS1, N, and F proteins were quantified by Western blot analysis. The values were normalized to endogenous GAPDH and expressed as a percentage of the normalized value for the same protein in the RSV wt sample at 18 hpi from the same experiment. The percentages from the three experiments were averaged and are shown in Figure 2. Because of the potential for experimental variability in these measurements of viral mRNAs and protein expression, we looked, in particular, for differences outside an arbitrary window ranging from 50% to 200% of the corresponding RSV wt 18 hpi value, which are indicated in Figure 2 with red boxes for <50% and blue boxes for >200%.

Among the control virus infections (RSV wt and ∆NS1), the levels of the NS1, N, and F mRNAs increased marginally from 18 hpi to 24 hpi (Figure 2). As expected, the ∆NS1 virus did not express NS1 mRNA. The levels of N and F mRNA expressed by ∆NS1 were less at both the 18 and 24 hpi time points compared to RSV wt, but the differences were not great (and within the arbitrary window described above), and likely reflected reduced replication of ∆NS1 compared to RSV wt. The levels of viral NS1, N, and F proteins approximated those of the corresponding mRNAs.

Among the mutant viruses, the levels of NS1 mRNA were generally similar to that of RSV wt (i.e., within 50% to 200% of RSV wt). In the case of viruses NS1 54-57-60, NS1 98-104st, and NS1 06 D (Figure 2), the mRNA values dipped below 50% of RSV wt, but only slightly. This likely reflected, at least in part, reduced virus replication. The levels of NS1 protein were similar to that of RSV wt except for NS1 11-13-15, NS1 06-09, NS1 58-66, NS1 54-57-60, NS1 98-104st, and NS1 06 D (Figure 2), which had markedly reduced levels. In particular, during infection with viruses NS1 06-09, NS1 98-104st, and NS1 06 D, the protein levels at 24 hpi were 4%, “Not detected”, and 8%, respectively. Interestingly, two of these three mutants had the substitution of leucine at position 6: NS1 06-09, involving L to A, and NS1 06 D, involving L to D (Figure 1).

The levels of N mRNA and protein were generally similar to those of RSV wt (i.e., were within the range of 50% and 200%). In several cases, the level of N protein dipped below 50%, but only marginally, and likely reflected reduced replication. Expression of F mRNA was also generally like that of RSV wt, and the same was true of the expression of F protein by mutants NS1 29 and NS1 98-109 (Figure 2). Unexpectedly, the remainder of the mutant viruses had substantially reduced expression of RSV F protein. For three of these mutants, namely, NS1 06 Q, NS1 06 R, and NS1 06 W, the levels of F protein were very low at 18 hpi but increased substantially by 24 hpi. For the other mutant viruses, the level of RSV F protein was low at both time points.

### 3.4. Host Cell mRNA and Protein Expression in A549 Cells during Infection with RSV NS1 Substitution Viruses

We analyzed the expression of several host cell mRNAs and/or proteins during infection of A549 cells with the mutant viruses—specifically, IFN-β, IFN-λ1, CCL5 (C-C motif chemokine ligand 5), also known as RANTES (regulated on activation, normal T-cell expressed and secreted), TNF-α (tumor necrosis factor alpha), and STAT2 (signal transducer and activator of transcription 2). A549 cells were inoculated at an MOI of 3.0 pfu/cell with the 12 mutant viruses in parallel with RSV-GFP and RSV-GFP/∆NS1 and incubated at 37 °C. From replicate cultures, cells were harvested at 18 and 24 hpi, and then processed for quantification of host mRNAs by qRT-PCR or host proteins by Western blot analysis. From additional cultures, the overlying tissue culture medium was harvested at 18, 24, and 36 hpi and analyzed by enzyme-linked immunosorbent assay (ELISA). The results are shown in Figure 3, with values <50% and >200% of RSV wt at 18 hpi indicated in red or blue boxes, respectively; green boxes indicate STAT2 levels that are <25% of uninfected cells.

As seen previously, ∆NS1 virus infection of A549 cells allowed substantially increased IFN-β mRNA expression (483% of RSV wt at 18 hpi), although the peak level of secreted IFN-β protein at 36 hpi by ELISA was like the peak of RSV wt at 24 hpi. IFN-β mRNA or protein expression was undetectable in mock-infected A549 cells. Among the mutant viruses, several phenotypes were observed. Surprisingly, for mutant viruses NS1 29 and NS1 98-109 (Figure 3), the expression of IFN-β mRNA and protein was reduced compared to RSV wt. For mutant NS1 47, there was an increase in the expression of IFN-β mRNA at 18 hpi compared to RSV wt, but this was reversed by 24 hpi, suggesting a build-up of NS1 activity with time. For mutants NS1 11-13-15, NS1 06-09, NS1 58-66, NS1 54-57-60, and NS1 98-104st, the levels of IFN-β mRNA and protein were substantially increased compared to RSV wt and, in several cases, was increased compared to the ∆NS1 virus, indicative of a substantial loss of inhibition of IFN expression. For mutants NS1 06 D, NS1 06 Q, NS1 06 R, and NS1 06 W, the levels of IFN-β mRNA and secreted IFN-β were generally similar to those of RSV wt, although the NS1 06 D mutant had higher levels of mRNA at 24 hpi and higher levels of IFN-β protein at all time points compared to RSV wt.

In contrast to IFN-β mRNA, expression of IFN-λ1 mRNA was somewhat higher for RSV wt than for ∆NS1, and levels of secreted IFN-λ1 protein were much higher for RSV wt than for ∆NS1. IFN-λ1 mRNA or protein expression was undetectable in mock-infected A549 cells. Among the mutant viruses, the peak expression levels of IFN-λ1 mRNA were like that of RSV wt, (i.e., within a window of <50% and >200%), with the exception of mutants NS1 06-09, NS1 54-57-60, and NS1 98-104st (Figure 3), which had much higher levels of IFN-λ1 mRNA. With regard to IFN-λ1 protein, three viruses had peak levels that were more than 200% that of RSV wt at 18 hpi—namely, NS1 29, NS1 98-109, and NS1 06 Q.

We previously showed that infection with RSV wt increased the expression of CCL5 and TNF-α proteins, and that this increase was less with mutants lacking either or both NS proteins [28]. In the present study, the expression of CCL5 and TNF-α mRNA in A549 cells infected with RSV wt rose to 574% and 380% of the 18 hpi expression levels, respectively (Figure 3), by 24 hpi, whereas expression of these same mRNAs at 24 hpi with the ∆NS1 virus was approximately the same for RSV wt infection at 18 hpi (Figure 3). CCL5 and TNF-α mRNA or protein expression were undetectable in mock-infected A549 cells. Some Group 1 members, NS1 29, NS1 98-109, NS1 06-09, NS1 58-66, and NS1 54-57-60 (Figure 3), as well as all Group 2 members, had levels of CCL5 mRNA expression at 24 hpi that were between 273% and 663% RSV wt 18 hpi. CCL5 mRNA expression remained lower (between 157% to 190% of RSV wt 18 hpi expression level) among the remaining members of Group 1, NS1 47, NS1 11-13-15, and NS1 98-104st, by 24 hpi (Figure 3).

Among the Group 1 viruses, expression of TNF-α by the NS1 mutant viruses NS1 29 and NS1 98-109 (Figure 3) was similar to that of RSV wt. In contrast, TNF-α expression by NS1 11-13-15, NS1 06-09, NS1 58-66, and NS1 54-57-60 was substantially lower at both 18 and 24 hpi. The remaining Group 1 viruses had intermediate values (Figure 3). Among all Group 2 viruses, the levels of TNF-α mRNA were low at 18 hpi, with all but NS1 06 D showing increased TNF-α mRNA expression by 24 hpi (Figure 3).

The RSV NS1 and NS2 proteins have been shown to degrade the STAT2 protein, resulting in reduced signaling through the type I IFN receptor [31,32,53]. To measure this activity for the NS1 mutant viruses, A549 cells were infected with an MOI of 3.0 as described previously, harvested at 18 and 24 hpi, and STAT2 expression quantified by Western blot analysis. These protein expression values were normalized to endogenous GAPDH and expressed as a percentage of the amount of STAT2 protein in uninfected A549 cells harvested 18 hpi. Two experiments were performed, and the mean values are shown in Figure 3.

In cells infected with RSV wt, STAT2 protein levels decreased to 3% of uninfected cells (Figure 3) by 24 hpi. With the ∆NS1 virus, STAT2 protein expression was 12% of uninfected A549 cells at 24 hpi (Figure 3), suggesting that RSV can mediate substantial destruction of STAT2 protein in the absence of the NS1 protein, probably due to NS2. The first three Group 1 viruses, NS1 29, NS1 98-109, NS1 47, and NS1 11-13-15 (Figure 3), resemble ∆NS1 virus in degrading STAT2 to levels 9–12% that of the uninfected 18 hpi control. The remaining Group 1, as well as all Group 2 viruses, had STAT2 levels of 15–33% that of the uninfected control, suggesting they were less effective degrading STAT2 protein than ∆NS1.

### 3.5. Host Cell Apoptosis in A549 Cells Infected with the RSV NS1 Substitution Viruses

The NS1 and NS2 proteins, individually and in combination, suppress the induction of apoptosis during RSV infection [11,20,54]. To assess apoptosis, A549 cells were infected with mutant and control viruses at an MOI of 3.0 pfu/cell as described in preceding experiments. Caspase3/7 activity was measured in quadruplicate at 12, 21, 24, 36, and 48 hpi using an assay in which a luminogenic compound is cleaved by caspase 3/7 into a substrate that can be used by luciferase to generate a luminescent signal [55]. The values from quadruplicate wells for each sample were averaged and expressed as a percentage of the value for RSV wt-infected cells at 12 hpi. Duplicate experiments were performed, and the mean values are shown in Figure 4, with values of >300% that of RSV wt at 12 hpi indicated with blue boxes.

The ∆NS1 virus had a reduced ability compared to RSV wt to delay apoptosis in A549 cells, as evidenced by higher signals than RSV wt at 12, 21, 24, and 36 hpi (Figure 4). NS1 29 and NS1 98-109 of Group 1, as well as NS1 06 Q, NS1 06 R, and NS1 06 W of Group 2, all exhibited a wt-like level of delayed/reduced apoptosis in A549 cells across all time points (Figure 4), indicating that this function of NS1 remained largely intact. The remaining members of Group 1 viruses, NS1 47, NS1 11-13-15, NS1 06-09, NS1 58-66, NS1 54-57-60, and NS1 98-104st, as well as NS1 06 D (Group 2), had earlier and higher levels of apoptosis compared to RSV wt (Figure 4), indicating a decrease in the ability of NS1 to delay/reduce apoptosis.

## 4. Discussion

We explored the novel approach of using machine learning and in silico predictions to identify potential attenuating mutations in the RSV NS1 protein structure, 5VJ2. In the mutational analysis of the 5VJ2 structure, the AUTO-MUTE 2.0 prediction algorithm combined knowledge-based four-body statistical potentials with machine learning to generate predictions of the effects of single amino acid substitutions on protein structure stability. Thermodynamic stability and free energy prediction programs were chosen to highlight specific substitution/locations in NS1, and these changes were evaluated in infectious RSV. To the best of our knowledge, this was the first example in which the AUTO-MUTE 2.0 prediction algorithm was used to make protein structure predictions that were then characterized for actual protein function in viable virus, of which a number of specific activities could be assessed.

Two different groups of predictions were evaluated. The mutations in Group 1 were chosen based on the predicted thermal stability change (Increased or Decreased) in combination with average ddG values among residue groups with specific functionality. The second group (Group 2) of mutations involved four different amino acid substitutions at a single position, amino acid 6. All four Group 2 substitutions were predicted as Decreased and exhibited a range of increasingly negative ddG scores (NS1 06 W, −0.58; NS1 06 Q, −1.26; NS1 06 D, −1.85; and NS1 06 R, −1.96). We investigated to what degree the prediction labels corresponded with the phenotypic characterization of viable virus.

The peak titer of the ∆NS1 virus in Vero cells was reduced by only 0.7 log_10_ pfu/mL in Vero cells, indicating that the activities of NS1 provided only a modest effect in this cell line that does not produce type I IFN in response to viral infection. This limited level of restriction may reflect the loss of activities of NS1 in antagonizing apoptosis and type III IFN, albeit of *Chlorocebus sabaeus* (African Green Monkey) origin. While all NS1 substitution mutants had at least one thermal stability prediction of Decreased, virus NS1 29 had the highest titer among the mutants and was essentially equal to RSV wt. The other NS1 substitution mutants had modest reductions of up to 1.1 log_10_.

As was already known, the differences in peak titer between RSV wt and ∆NS1 was much greater in A549 cells, which are able to make human type I and III IFN in response to viral infection. Thus, A549 cells can be used in assays for NS1 function. Among the three Group 1 viruses least reduced in peak replication, all had small ddG predictions that may better explain this limited reduction in virus growth than the thermal stability labels. However, the third virus, NS1 98-109, had a Decreased thermodynamic stability prediction despite a small positive ddG prediction. This possibly reveals some limitations of machine learning to separate small, continuously scaled sets of values (ddG) into groups (Increased or Decreased), or of making predictions in less ordered protein structures found in the vicinity of NS1 98-109. Conversely, the remaining members of Group 1 viruses demonstrate a greater loss of peak viral replication. This suggests that these substitution mutations with larger ddG values may represent better machine-learning predictions (resulting in reduced NS1 function and peak viral replication). Finally, in A549 cells, titers of Group 2 viruses were similar, suggesting that peak virus titers may not be sensitive in revealing subtle differences due to these single amino acid replacements. Nevertheless, the overall lower titers of the Group 2 viruses compared to wt RSV are generally consistent with the predicted destabilizing nature of these mutations. All NS1 substitution mutants had peak titers that were intermediate between RSV wt and ∆NS1, consistent with the expectation that the effects of point mutations generally would be less than that of a complete gene deletion.

We also evaluated the accumulation of viral NS1, N, and F mRNAs and proteins in infected A549 cells. A previous study with RSV minigenomes suggested that NS1 protein is a potent inhibitor of RSV transcription and RNA replication [56]. However, effects of NS1 on RNA synthesis by complete genomes have not been described and were not observed in the present study. The deletion of NS1 was associated with only a marginal reduction in viral transcription and viral protein synthesis that may more likely be related to decreased viral replication. The two NS1 substitution mutants that most closely resembled RSV wt for growth in A549 cells, NS1 29 and NS1 98-109 (with predictions of Decreased and Increased/Decreased, respectively), also most closely resembled RSV wt regarding the accumulation of NS1, N, and F mRNAs and proteins. The levels of accumulation of viral mRNAs and proteins for the other NS1 substitution mutants generally were similar to, or marginally less than, that of RSV wt, with two exceptions. First, six of the mutants (NS1 11-13-15, NS1 06-09, NS1 58-66, NS1 54-57-60, NS1 98-104st, and NS1 06 D) exhibited drastic reductions in the accumulation of NS1 protein but not NS1 mRNA. The substantially lower levels of protein compared to mRNA suggested the possibility that these mutations destabilized the NS1 protein sufficiently to cause increased degradation. Alternatively, one or more of these mutations may have reduced the efficiency of binding by the monoclonal antibody used in the Western blots, so that the protein might be present, but the efficiency of detection reduced. A second unexpected finding was that, for all the mutants other than the wt-like NS1 29 and NS1 98-109 mutants, the accumulation of F protein was drastically reduced at 18 hpi compared to RSV wt. In several cases (NS1 06 Q, NS1 06 R, and NS1 06 W), the levels of F increased substantially by 24 h, suggesting that the expression of F was delayed but subsequently approached that of RSV wt. In the other cases, the levels of F remained reduced at 24 hpi. The basis for the lower levels of F relative to NS1 and N is unknown. One possibility is that these mutants have an increased gradient of transcription, although comparable effects on transcription have not previously been associated with the NS1 protein. Further study is needed given these observations.

The NS1 substitution viruses were also characterized based on several aspects of the host response in A549 cells. Expression of IFN-β is an important early host response to RNA virus infection, as well as a main target of RSV NS1. As previously shown, the expression of IFN-β mRNA was substantially increased when NS1 was deleted. The expression of IFN-β mRNA in response to NS1 29 and NS1 98-109 was somewhat reduced compared to that of wt RSV, raising the possibility of increased NS1 function associated with the prediction of increased thermal stability. IFN-β expression was undetectable in mock-infected A549 cells. Most of the other Group 1 members exhibited substantially elevated IFN-β mRNA expression, consistent with reduced inhibition by NS1 protein. Two of these mutants, namely, NS1 06-09 and NS1 98-104st, were among the ones noted above with very low levels of NS1 protein. Therefore, the elevated levels of IFN-β mRNA might be due to destabilization and degradation of NS1 rather than mutagenesis of residues directly involved in IFN antagonism. In contrast, the Group 2 viruses had very low levels of IFN-β mRNA, similar to RSV wt, suggesting that residue 6 was not important to IFN antagonism activity of RSV NS1 protein. Among the Group 2 mutants, NS1 06 D had the most negative ddG value (Figure 1) and the lowest level of expression of NS1 protein, which could account for the increased IFN-β expression compared to RSV wt. In general, the peak viral titers in A549 cells appeared to be inversely related to the expression of IFN-β mRNA, suggestive of direct and/or indirect restriction of viral replication by this cytokine.

IFN-λ1, a type III IFN, is another important mediator of the host innate immune response to virus infection that has overlap with the pathways activated by IFN-β, a type I IFN. While each IFN type activates different receptor complexes, both lead to similar antiviral, apoptotic, and immunomodulatory effects in infected cells. A major difference is that type III IFN receptor complexes are primarily found on epithelial cells and, therefore, thought to provide antiviral protection to epithelial surfaces in particular. The expression of IFN-λ1 mRNA in A549 cells infected with RSV wt or ∆NS1 virus almost doubled between 18 hpi and 24 hpi but remained virtually static over the same time period during NS1 29 and NS1 98-109 infections. IFN-λ1 expression is undetectable in mock-infected A549 cells. Most other Group 1 members expressed levels of IFN-λ1 mRNA similar to that of RSV wt except for NS1 06-09 and NS1 54-57-60 which, by 24 hpi, expressed much higher levels. These same two viruses were among the highest inducers of INF-β mRNA.

Proinflammatory chemokines and cytokines such as CCL5 may contribute to stimulating host immunity and restricting RSV replication, but also may contribute to RSV disease [57,58]. Consistent with previous work [22], RSV wt virus induced CCL5 and TNF-α, whereas ∆NS1 virus induced substantially lower levels of both cytokines. Three of the NS1 substitution mutants were relatively inefficient at inducing CCL5 mRNA (NS1 47, NS1 11-13-15, and NS1 98-104st), while the others resembled RSV wt with robust induction of CCL5 mRNA.

TNF-α, another chemokine whose expression is increased upon RSV wt infection [57,58], was induced less strongly by the ∆NS1 deletion mutant both in previous work [22] and the present study (Figure 3). The expression of TNF-α was also reduced for most of the NS1 substitution mutants with the highest level of expression of IFN-β mRNA (e.g., NS1 11-13-15, NS1 06-09, NS1 58-66, and NS1 54-57-60) being associated with the lowest levels of TNF-α mRNA. The basis for this effect is not known.

STAT2 has been reported to be degraded by NS1 and NS2 independently and together during RSV infection [31,32,53]. Compared to mock-infected A549 cells, NS1 29, NS1 98-109, and NS1 47 viruses exhibited substantial reductions in STAT2 accumulation at 18 and 24 hpi that generally were intermediate between RSV wt and RSV ∆NS1. The remaining members of Group 1 viruses exhibited a somewhat lesser reduction in STAT2 protein expression at 18 hpi and in most cases at 24 hpi. A final observation involved the discrepancy between NS1 98-104st and ∆NS1. Based on the lack of detectable NS1 protein in both samples, it was surprising to not see an equivalent reduction in STAT2 expression for these viruses. However, as mentioned previously, this may reflect a lack of binding by the NS1 monoclonal antibody rather than an actual loss of protein.

Apoptosis is a host cell defense mechanism that can reduce virus yield, as well as facilitate antigen presentation. During RSV infection of tissue culture cells, the induction of apoptosis is slowed and reduced by NS1 (and NS2), resulting in an increase in virus yield [11,20,54]. Measuring caspase 3/7 activity, which precedes the irreversible apoptotic process, showed that this activity occurred earlier and at a higher level in cells infected with RSV ∆NS1 compared to RSV wt. NS1 29 and NS1 98-109, as well as the Group 2 viruses NS1 06 Q, NS1 06 R, and NS1 06 W, resembled RSV wt in the levels of caspase 3/7 activity, whereas the other mutants had substantially higher levels of caspase 3/7 activity (Figure 4).

**Figure 6:**
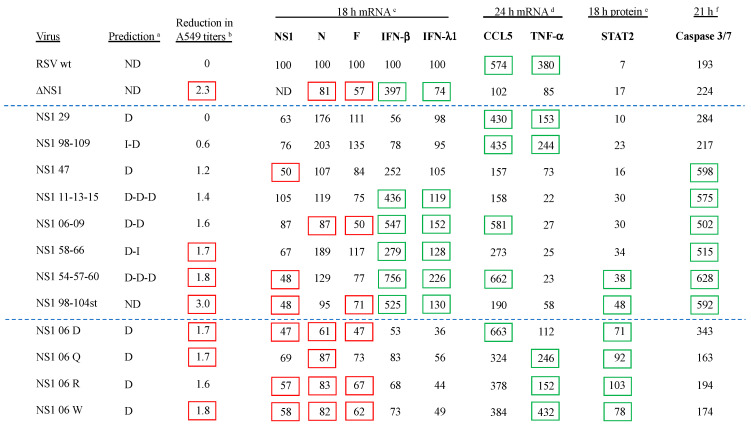
Summary of properties of the NS1 substitution viruses. ^a^ Thermal stability predictions (individually for each amino acid) from Figure 1. I = Increased stability; D = Decreased stability; ND = Not done. ^b^ A549 log_10_ peak titer reduction versus RSV wt (from Figure 2). ^c^ NS1, N, and F mRNA (from Figure 2) and IFN-β and IFN-λ1 mRNA (from Figure 3) expression levels at 18 hpi. ^d^ CCL5 and TNF-α mRNA (from Figure 3) expression levels at 24 hpi. ^e^ STAT2 protein expression (from Figure 3). ^f^ Caspase 3/7 activity (from Figure 4). The greatest increases or decreases, are boxed in green and red, respectively. Dashed blue lines separate the control virus results (top) from Group 1 viruses (middle) and Group 2 viruses (bottom).

Therefore, in general, measuring cell processes that were expected to be influenced by NS1 suggested that most viruses with a prediction of decreased NS1 thermodynamic stability generated assay outcomes that would be expected if NS1 function is compromised or missing. We sought to illustrate the use of these findings to presumptively identify promising vaccine candidates from the set of computationally predicted NS1 substitution mutants. For this comparison, we simplified the data from Figure 1, Figure 2, Figure 3 and Figure 4, resulting in Figure 6. The 12 viruses were designated as having increased (I), decreased (D), or mixed (e.g., D-D-I) thermal stability based on the scores for the individual amino acid substitutions from Figure 1. The peak titers in A549 cells compared to RSV wt are indicated, as are the levels of NS1, N, and F mRNAs relative to RSV wt, from Figure 2. The levels of mRNA for IFN-β, IFN-λ1, CCL5, and TNF-α relative to RSV wt are shown, as are the levels of STAT2 protein and caspase 3/7 activity, from Figure 3 and Figure 4. For the mRNAs and proteins, a single time point is shown. The six or seven greatest increases or decreases, as appropriate, are boxed in green and red, respectively.

For example, the mutant NS1 11-13-15 had a prediction of decreased thermodynamic stability (D-D-D for the amino acid substitutions at positions 11, 13, and 15, respectively) for NS1 and a moderate level of restriction (1.4 log_10_ reduction), but maintained a substantial expression of F mRNA (75%), which encodes the major RSV protective antigen. This virus induced high levels of IFN-β (436%) and IFN-λ1(119%), which can be immunostimulatory. It induced moderate levels of CCL5 (158%) and TNF-α (22%) mRNA, which can be either immunostimulatory or immunopathogenic depending on contextual expression levels. There was a substantial level of STAT2 (38%) and a strong apoptotic response (575%), which can be immunostimulatory (Figure 6). Thus, the mutations in this virus are of interest for further development. In comparison, mutant NS 06 Q, with a prediction of decreased stability (D), also had a moderate level of restriction (1.7 log_10_ reduction) and moderate levels of expression of viral mRNAs, but, in contrast to NS1 11-13-15, it induced low levels of IFN-β (83%) and IFN-λ1 (56%), and relatively high levels of CCL5 (324%) and TNF-α (246%). It also had a relatively high level of STAT2 (92%) and a moderate level of caspase activity (163%) (Figure 6). Thus, this would be another candidate of interest for pre-clinical development and might yield information on the contribution of IFNs and inflammatory cytokines like CCL5 to protective immunity and immunopathogenesis.

## 5. Conclusions

In conclusion, machine-learning predictions identified numerous mutations that affected NS1 functions and provided attenuating mutation candidates. These results support the use of machine-learning prediction software as a simple, versatile, and rapid method for identifying mutations that perturb a protein structure to varying extents and potentially provide a more efficient means of developing attenuating mutations. These results examining the RSV NS1 protein suggest that this type of analysis is well-suited for the investigation of any protein of unknown structure and unknown function.

## Data Availability

The original contributions presented in the study are included in the article/Supplementary Material, further inquiries can be directed to the corresponding authors.

## Supplementary Materials

The following supporting information can be downloaded at: https://www.mdpi.com/article/10.3390/v16060821/s1, File S1: 5vj2_m0_all.txt; File S2: ddG_5vj2_m2_all_out.txt; File S3: model1_m0_wo_68.txt; Table S1: All_NS1_mutation_changes_in_5vj2_m0_all.xlsx; Table S2: Vero and A549 timecourse table.xlsx; Table S3: Vero and A549 timecourse.xlsx; Table S4: Growth level calculations.xlsx; Table S5: Custom RSV TaqMan Assays.txt; Table S6: Virus RT_PCR table.xlsx; Table S7: Host RT_PCR table.xlsx; Table S8: Virus Protein table.xlsx; Table S9: RSV N and F protein expression percentage.xlsx; Table S10: RSV N and F and STAT2 protein expression percentage.xlsx; Table S11: Host Protein STAT2 table.xlsx; Table S12: Host ELISA table_01.xlsx; Table S13: Apoptosis table 01.xlsx; Figure S1: NS1 mutant NS1 western blots.pdf; Figure S2: NS1 mutant STAT2 western blots.pdf; Figure S3: NS1 mutant N western blots.pdf; Figure S4: NS1 mutant F western blots.pdf.
