# Supplementary figures and images for: Respiratory Syncytial Virus Vaccine Design Using Structure-Based Machine-Learning Models
Source: Viruses. 2024 May 22;16(6):821. doi: 10.3390/v16060821 (PMC11209532; doi:10.3390/v16060821)

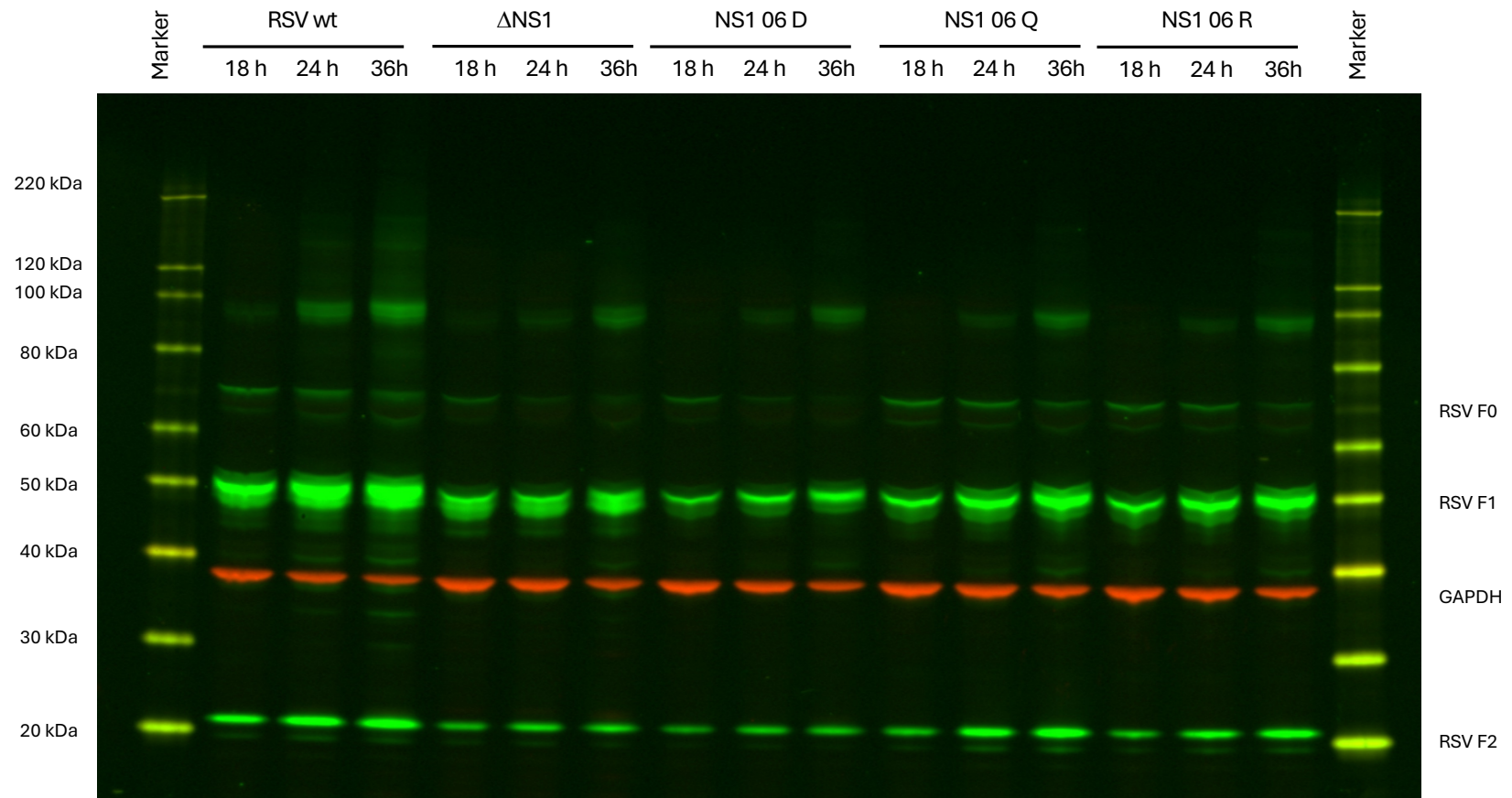

RSV F expression in A549 (MOI = 3)

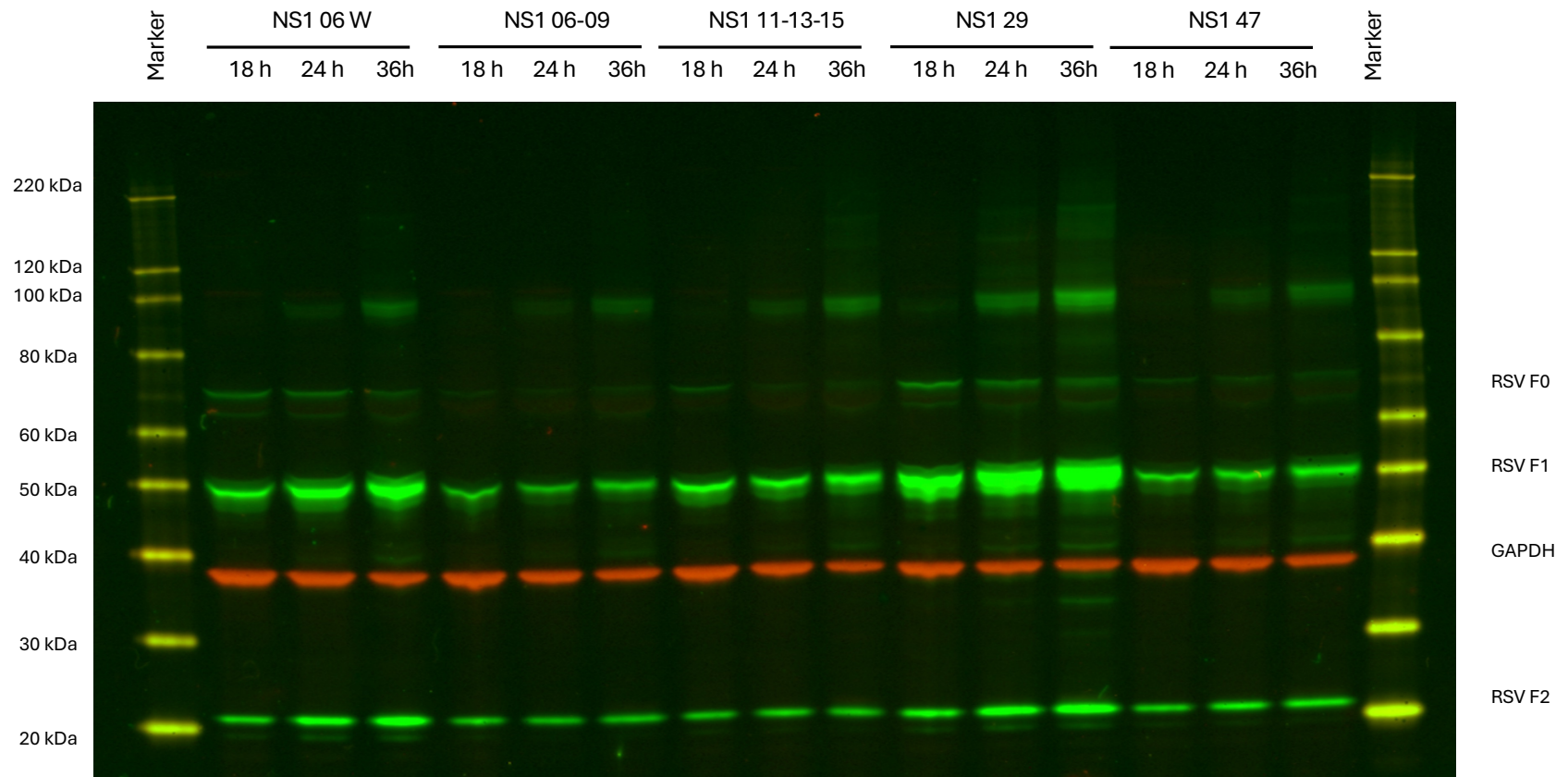

RSV F expression in A549 (MOI = 3)

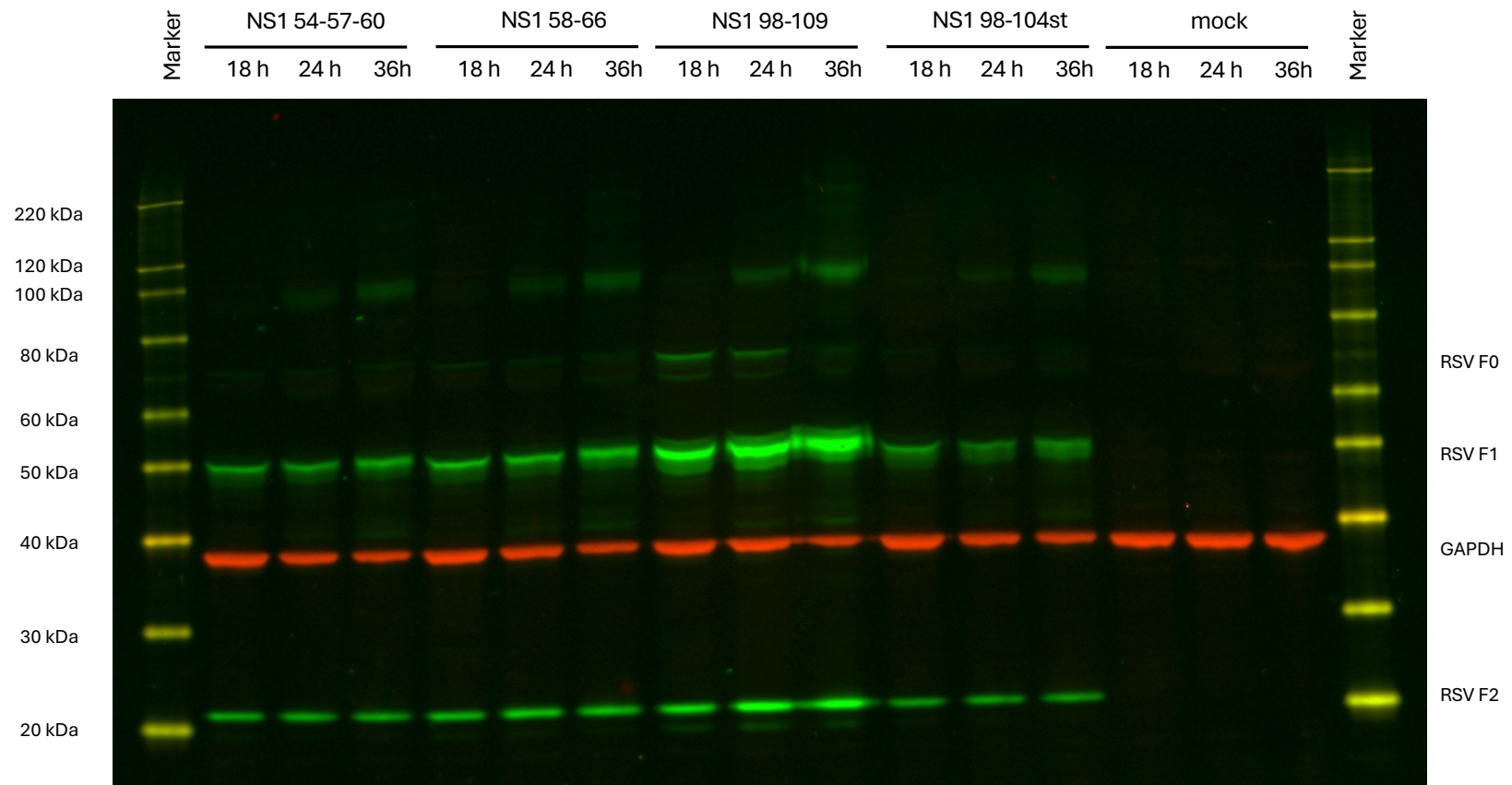

RSV F expression in A549 (MOI = 3)

Supplement: Supplementary file 1 [file viruses-16-00821-s001.zip › NS1 mutant F western blots.pdf]

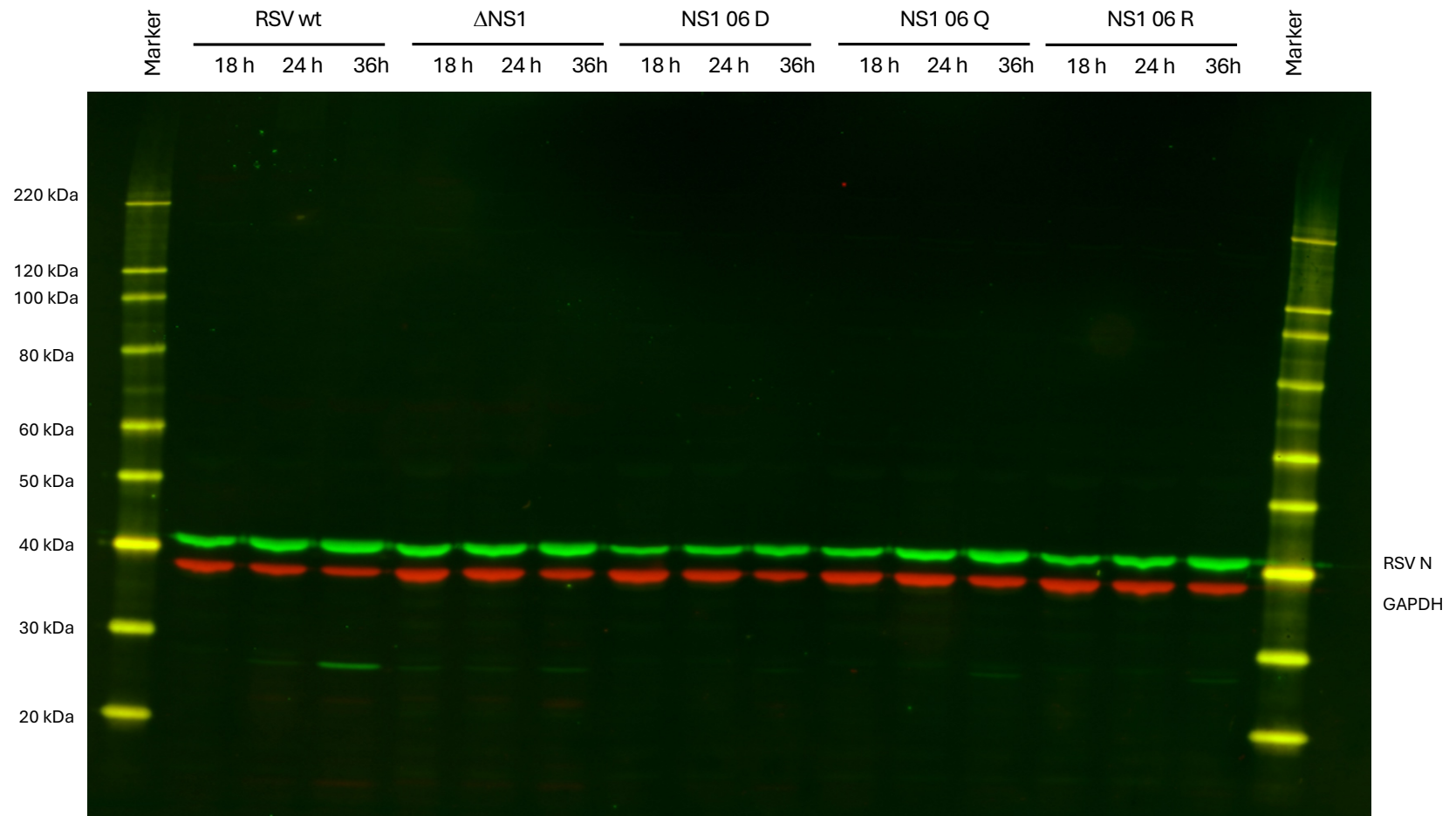

RSV N expression in A549 (MOI = 3)

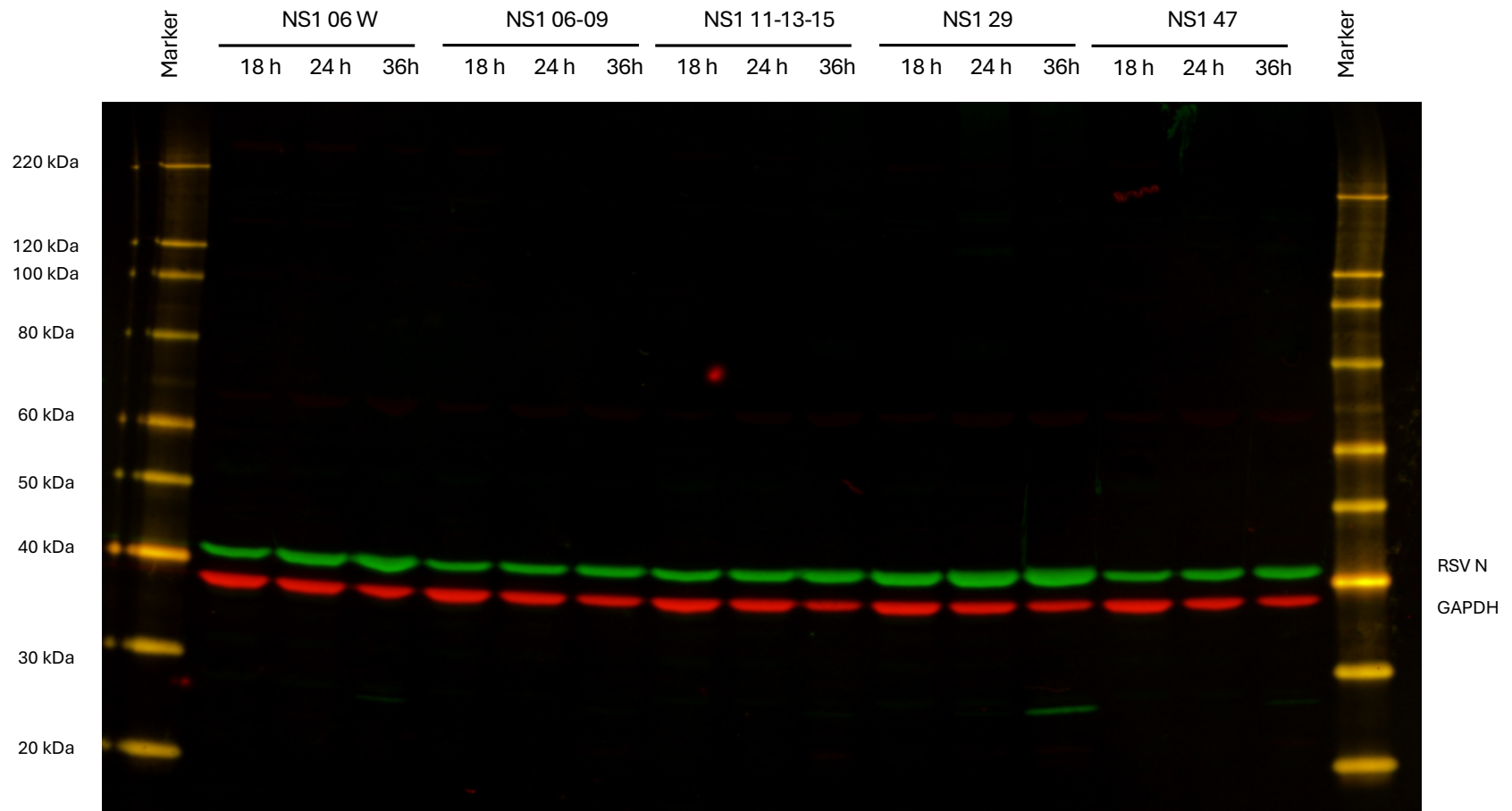

RSV N expression in A549 (MOI = 3)

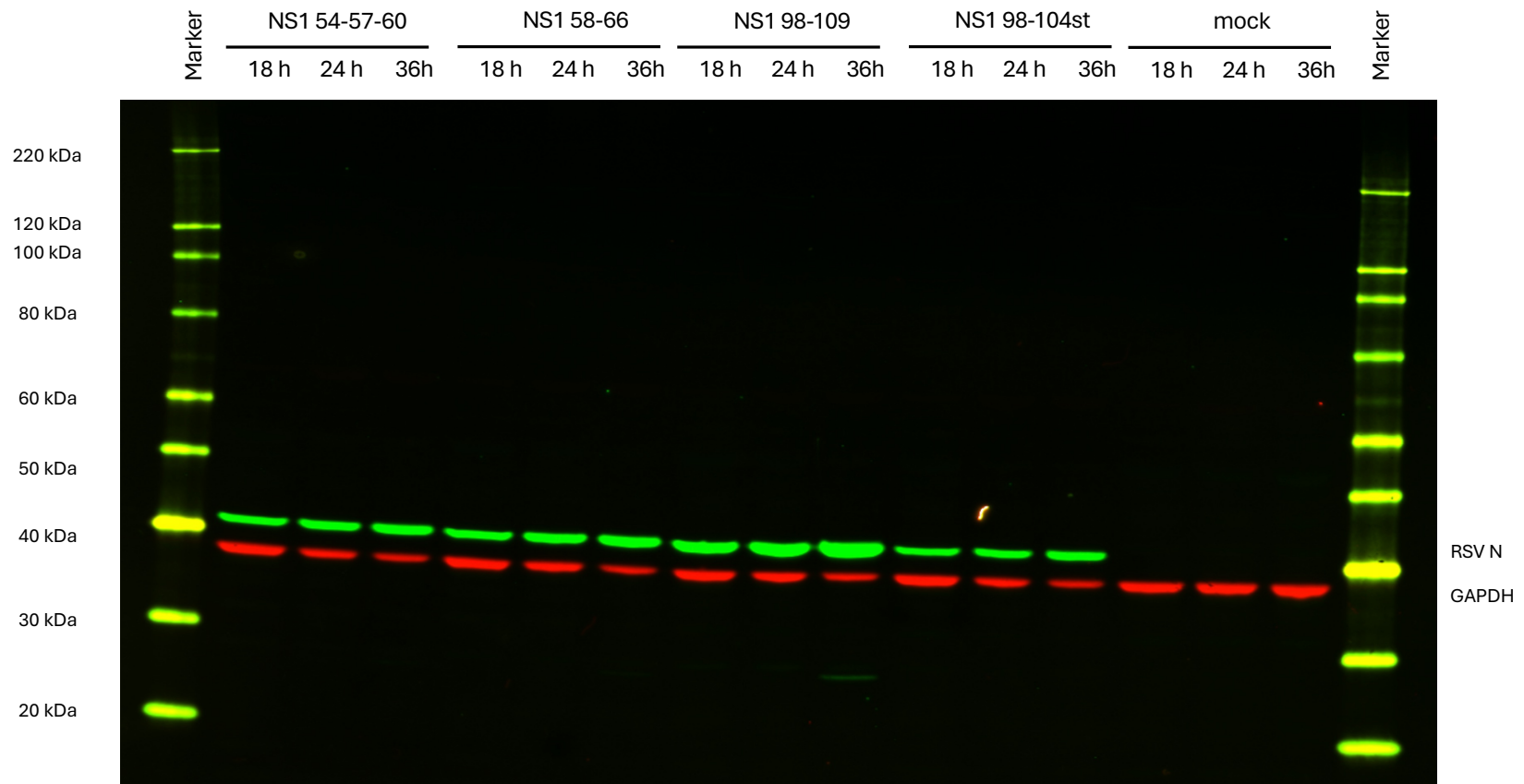

RSV N expression in A549 (MOI = 3)

Supplement: Supplementary file 1 [file viruses-16-00821-s001.zip › NS1 mutant N western blots.pdf]

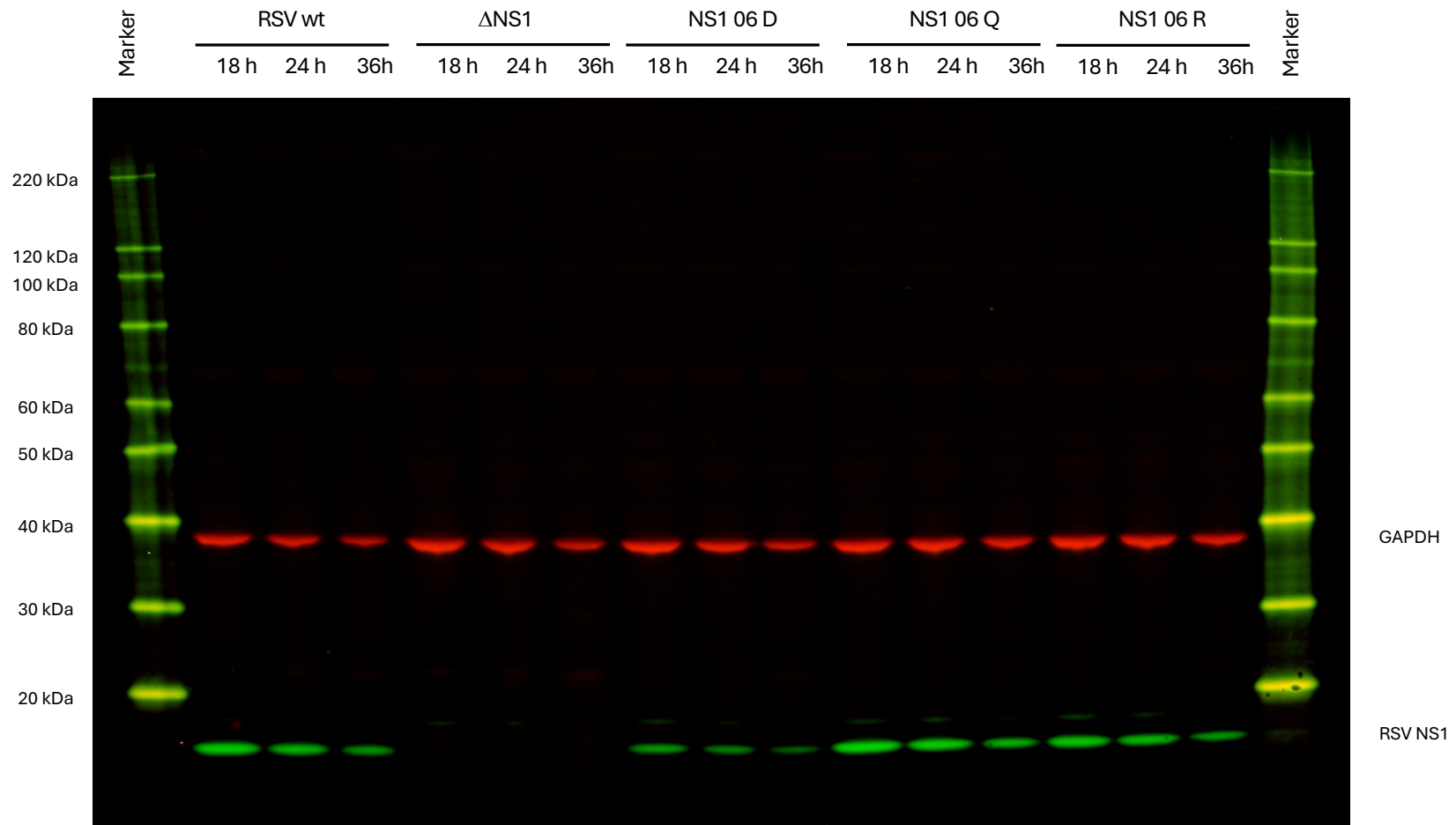

RSV NS1 expression in A549 (MOI = 3)

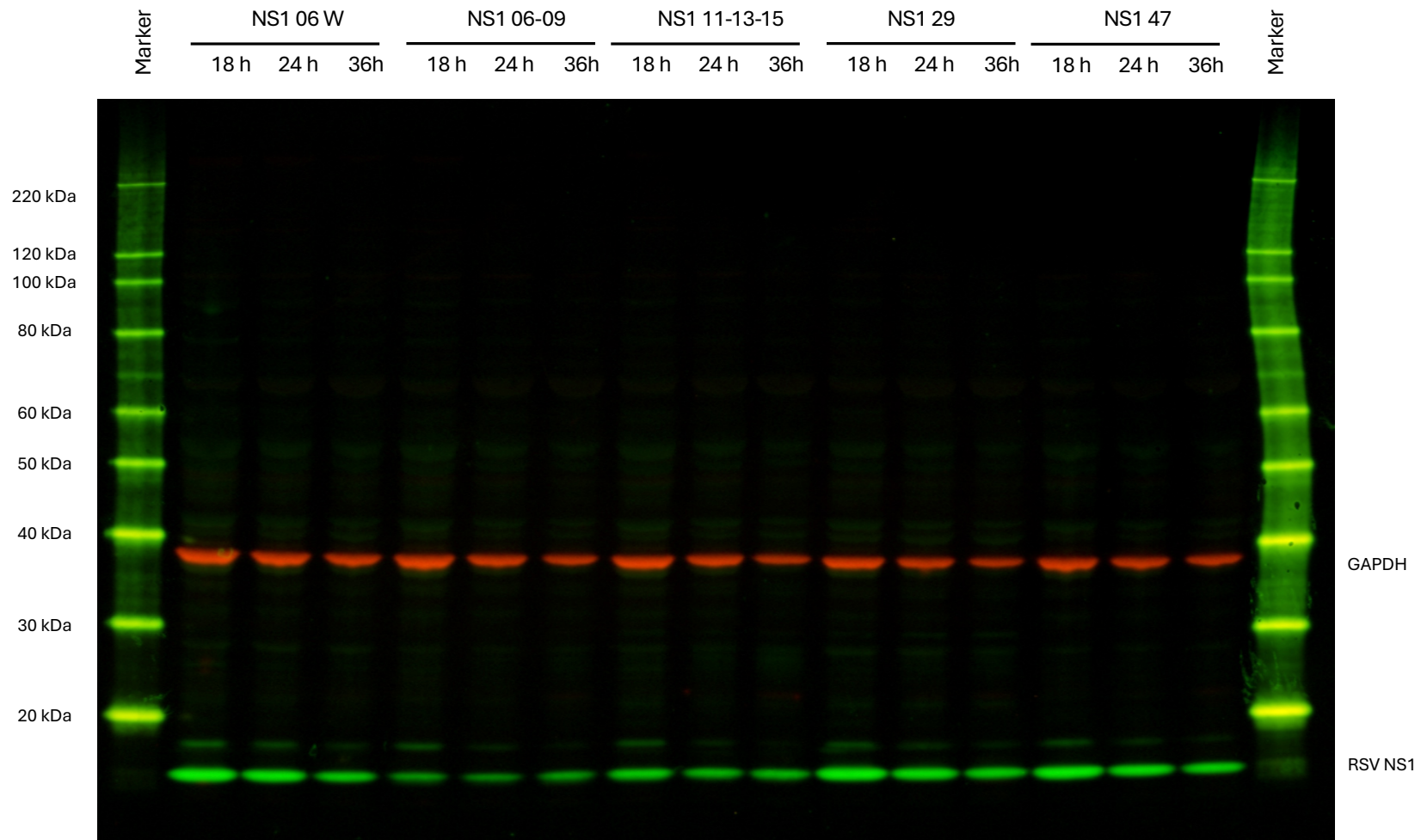

RSV NS1 expression in A549 (MOI = 3)

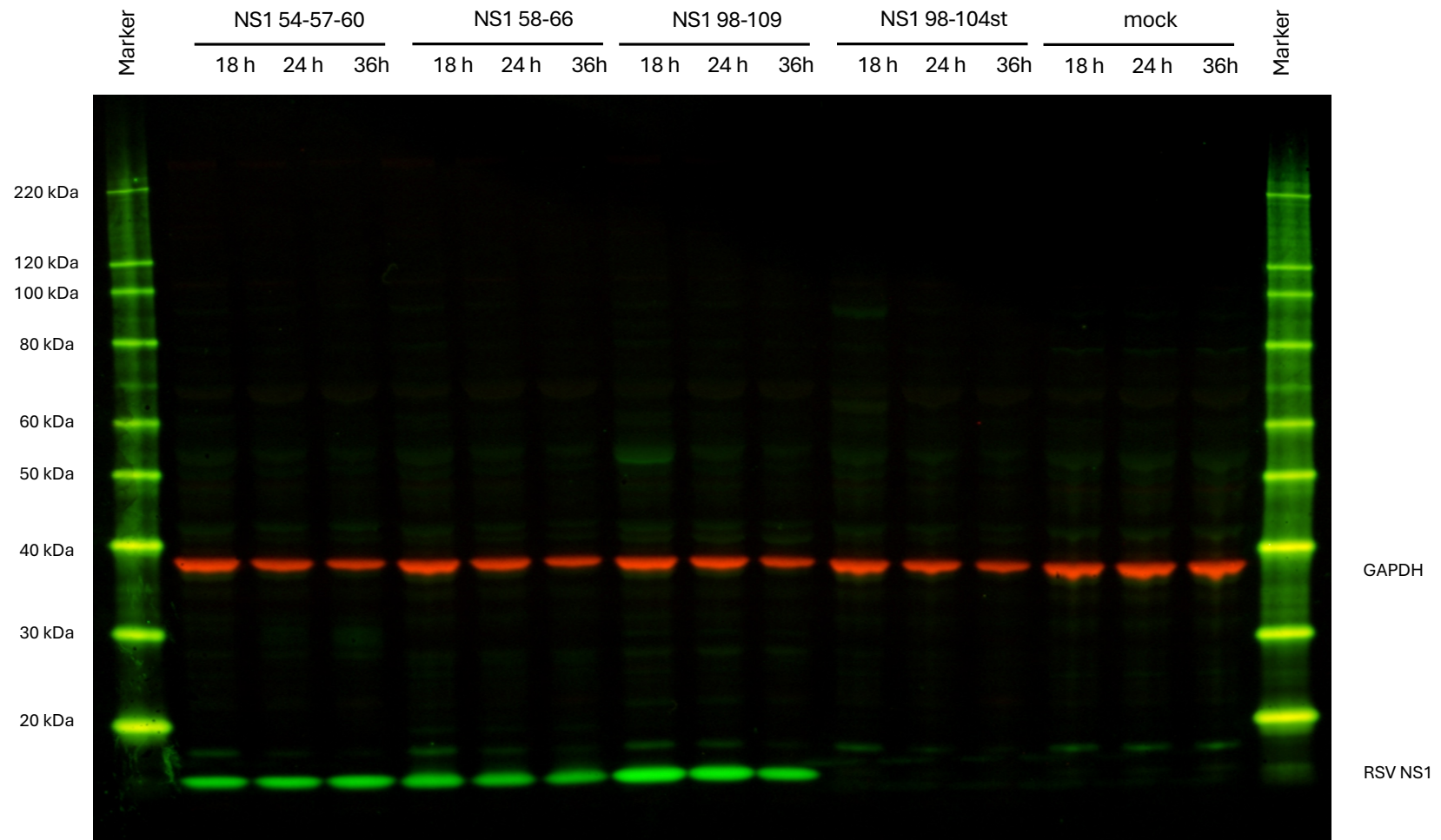

RSV NS1 expression in A549 (MOI = 3)

Supplement: Supplementary file 1 [file viruses-16-00821-s001.zip › NS1 mutant NS1 western blots.pdf]

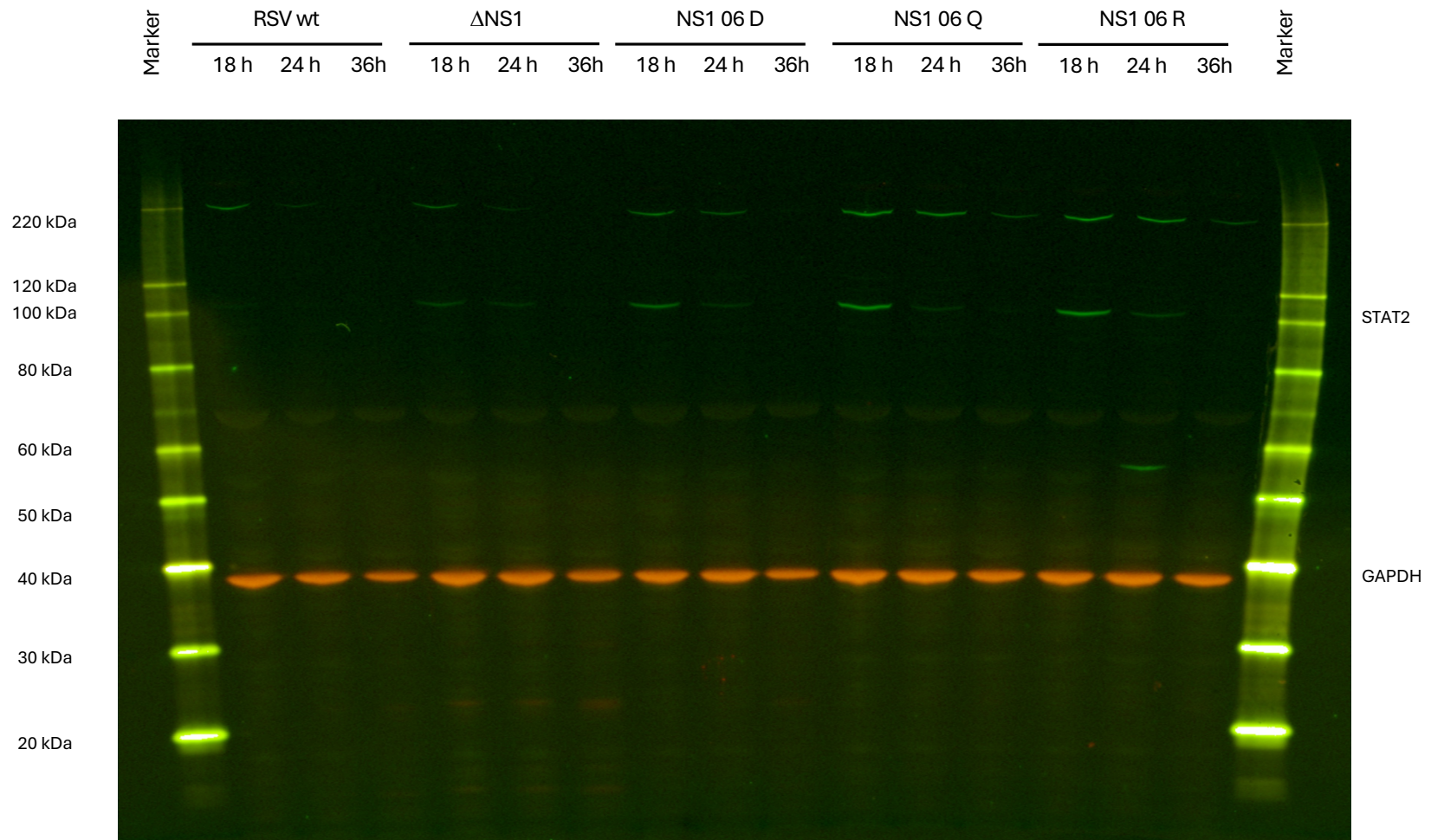

STAT2 expression in A549 (MOI = 3)

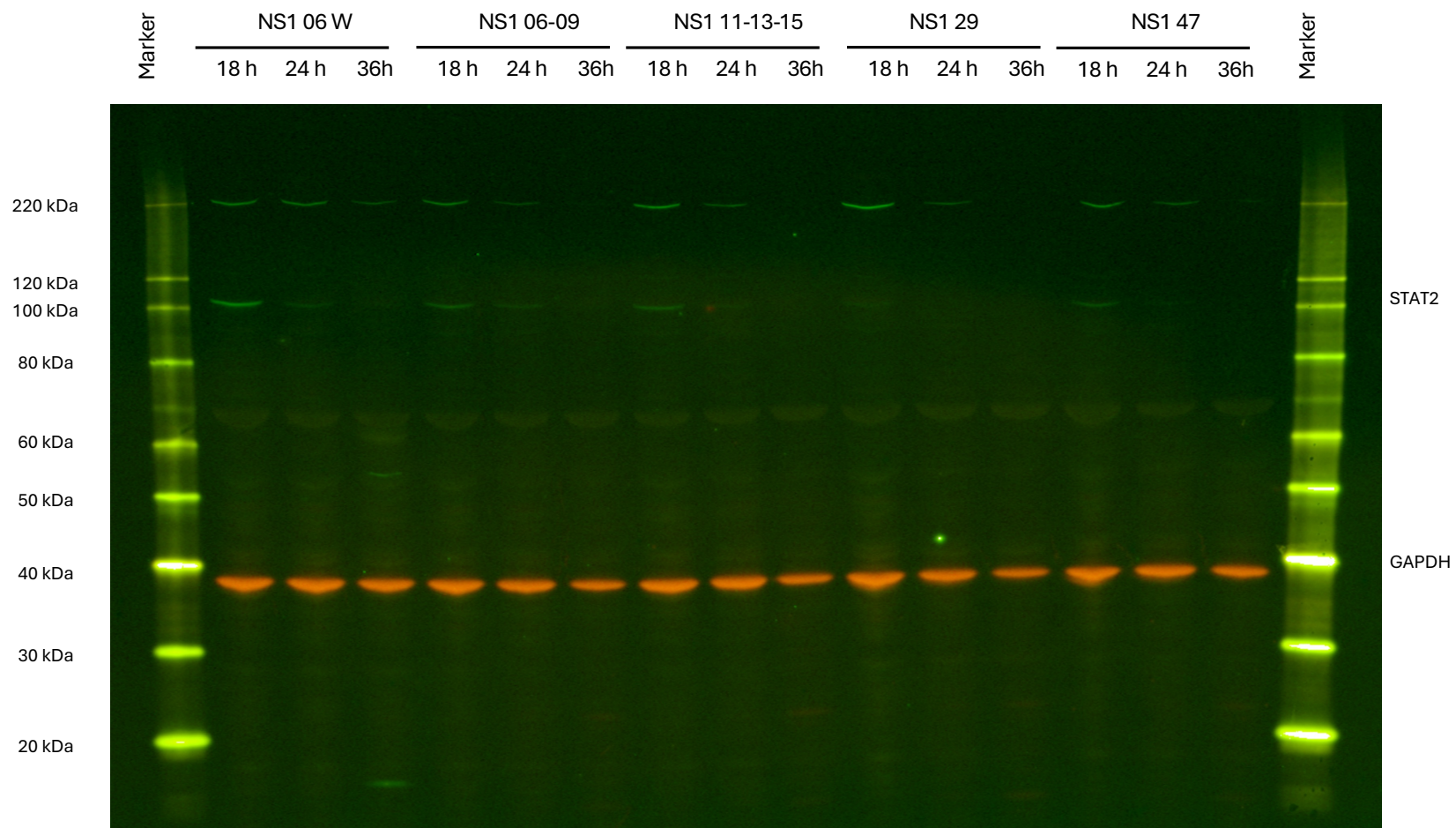

STAT2 expression in A549 (MOI = 3)

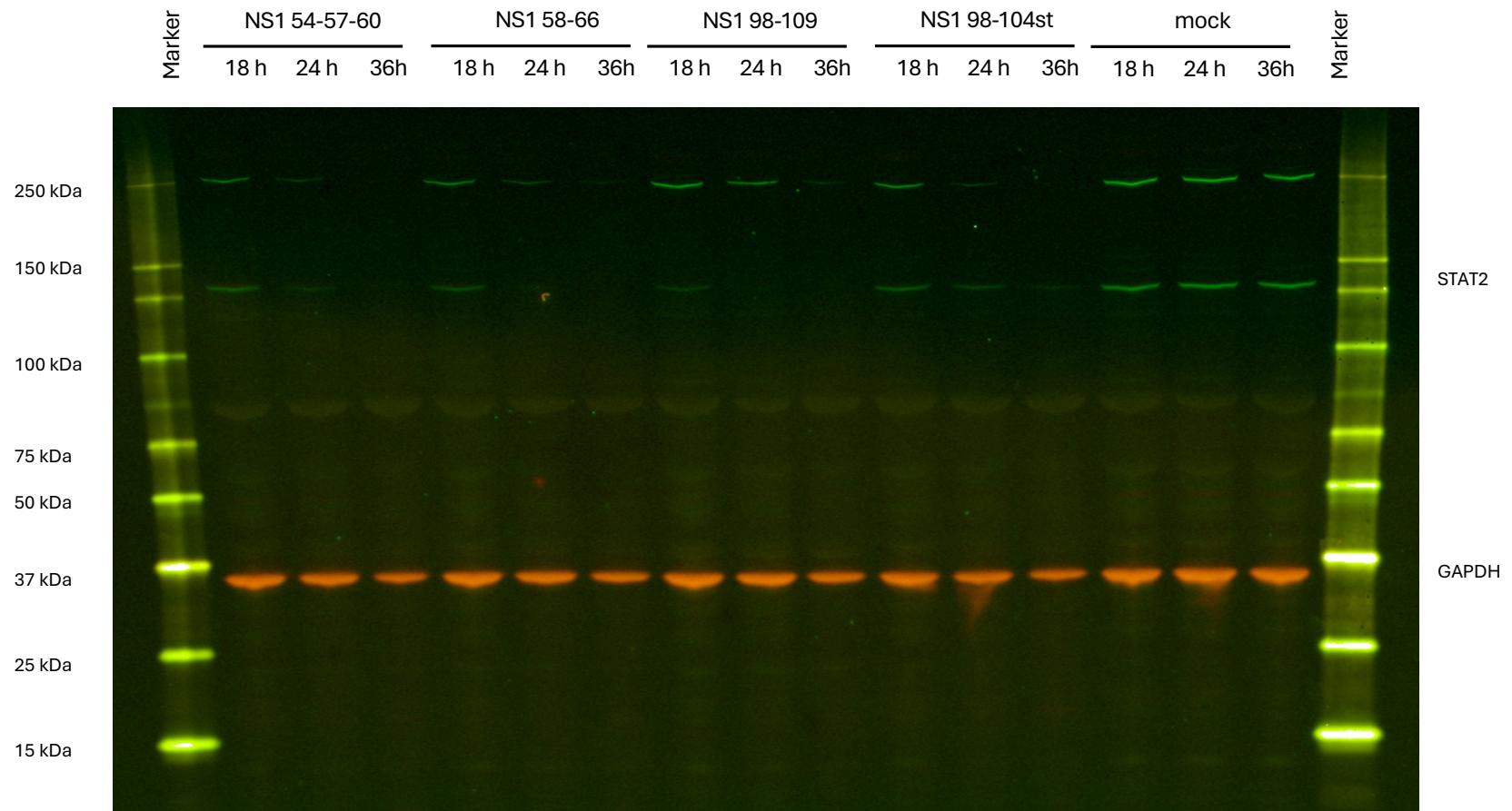

STAT2 expression in A549 (MOI = 3)

Supplement: Supplementary file 1 [file viruses-16-00821-s001.zip › NS1 mutant STAT2 western blots.pdf]
